# Dual Salt Cation-Swing Process for Electrochemical CO_2_ Separation

**DOI:** 10.1021/acscentsci.3c00692

**Published:** 2023-08-30

**Authors:** Fang-Yu Kuo, Sung Eun Jerng, Betar M. Gallant

**Affiliations:** † Department of Chemical Engineering, Massachusetts Institute of Technology, Cambridge, Massachusetts 02139, United States; ‡ Department of Mechanical Engineering, Massachusetts Institute of Technology, Cambridge, Massachusetts 02139, United States

## Abstract

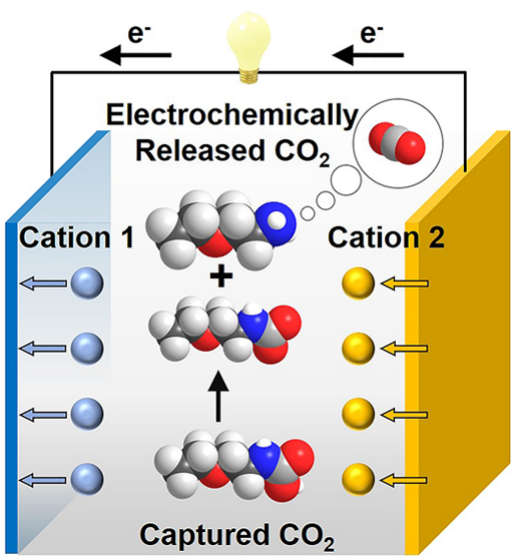

Electrochemical CO_2_ separations, which use electricity rather than thermal energy to reverse sorption of CO_2_ from concentrated point sources or air, are emerging as compelling alternatives to conventional approaches given their isothermal, ambient operating conditions, and ability to integrate with renewable energy inputs. Despite several electrochemical approaches proposed in previous studies, further explorations of new electrochemical CO_2_ separation methods are crucial to widen choices for different emissions sources. Herein, we report an electrochemical cation-swing process that is able to reversibly modulate the CO_2_ loading on liquid amine sorbents in dimethyl sulfoxide (DMSO) solvent. The process exploits a reversible carbamic acid-to-carbamate conversion reaction that is induced by changing the identity of Lewis acid cations (*e.g*. K^+^, Li^+^, Ca^2+^, Mg^2+^, and Zn^2+^) coordinated to the amine-CO_2_ adduct in the electrolyte. Using ethoxyethylamine (EEA) as a model amine, we present NMR-based speciation studies of carbamic acid-to-carbamate conversion as a function of amine/salt concentrations and cation identity. The reaction is further probed using gas-flow reaction microcalorimetry, revealing the energetic driving forces between cations and the amine-CO_2_ adduct that play a key role in the described re-speciation. A prototype electrochemical cell was further constructed comprising a Prussian white (PW) potassium (K^+^) intercalation cathode, zinc (Zn) foil anode, and EEA/DMSO electrolyte containing a dual KTFSI/Zn(TFSI)_2_ salt. A low CO_2_ separation energy of ∼22–39 kJ/mol CO_2_ (0.1–0.5 mA cm^–2^) was achieved with a practical CO_2_ loading delta of ∼0.15 mol CO_2_/mol amine. Further optimizations in electrolyte design and cell architectures toward continuous CO_2_ capture-release are expected to enhance rate performance while retaining favorable separation energies.

## Abstract 2

A newly developed electrochemical process reversibly modulates CO_2_ loading on liquid amine sorbents at room temperature by controllably altering the Lewis acid cation populations in the electrolyte upon cell discharge or charge.

## Introduction

Mitigating anthropogenic carbon dioxide (CO_2_) emissions from fossil fuel combustion requires the development of improved CO_2_ separation technologies with low energy requirements, durability, and versatility. While renewables-based electrification of key industries like power generation must be prioritized, starkly fewer solutions are immediately available for hard-to-decarbonize sectors such as heat- and CO_2_-intensive manufacturing (*e.g*. cement, steel, and chemical production).^1−5^ The incumbent CO_2_ separation process, which is largely matured, utilizes aqueous amine solutions that operate via a temperature swing between ∼40 °C on capture and 120–130 °C upon regeneration. This process still faces long-standing challenges such as high energy requirements (>80 kJ/mol CO_2_) for amine regeneration,^6−8^ amine degradation at high temperature, corrosion, aerosol production, high capital and operating costs, among others,^9−11^ limiting practical deployment. In this context, electrochemical separation approaches are gaining increasing attention due to several advantages, including ambient operating conditions, amenability toward direct integration with renewables as the energy input, and potential for modular designs.^12−17^ Salient examples of electrochemical CO_2_ separation processes include electrochemically mediated amine regeneration (EMAR),^17−19^ direct redox of organic sorbent molecules (e.g., quinones,^14,20,21^ sp^2^-nitrogen base,^22,23^*etc*.), and pH swing^16,24,25^ methods achieved through water electrolysis or use of bipolar membranes. Demonstrated energy requirements for these electrochemical processes at the cell level range from 30 to 100 kJ mol^–1^ CO_2_, evidencing their potential competitiveness with thermal-swing approaches if scaling can be achieved.^18−25^

While most CO_2_ capture processes—including those with amines—employ aqueous solutions, there has been growing consideration of conducting both conventional and electrochemical separation in nonaqueous media.^27−31^ One advantage of doing so is the higher amine-CO_2_ loading that can be reached by exploiting the tendency of some nonaqueous solvents to bind chemisorbed CO_2_ as neutrally charged carbamic acid (RNHCOOH, 1 mol CO_2_/mol amine) compared to a maximum of ∼0.5 mol CO_2_/mol amine (ionic ammonium carbamate, RNH_3_^+^ RNHCOO^–^) favored in primary/secondary amine aqueous solutions.^27^ The higher loading in nonaqueous solution has been attributed to hydrogen bonding stabilization between solvent and carbamic acid,^27^ whereas water does not effectively stabilize the acid form. For nonaqueous amine solutions that favor carbamic acid, sustaining an electrochemical process requires inclusion of a supporting electrolyte salt to impart ionic conductivity to the electrolyte, which can interact with amine species in solution and lead to speciation changes.^32^ For instance, we previously observed by ^1^H NMR spectroscopy that, while DMSO-based amine solutions favor carbamic acid, addition of a supporting Li^+^-based salt induces a substantial speciation change from ∼100% carbamic acid to a lower limit of ∼50% carbamate/∼50% ammonium (2 RNHCOOH + LiClO_4_ → RNH_3_^+^ + RNHCOO^–^Li^+^ + CO_2_ + ClO_4_^–^). This speciation change implies release of one CO_2_ from every two amine molecules to achieve the stoichiometric charge rebalancing triggered by the favorable interaction of Li^+^ with RNHCOO^–^, though direct evidence of CO_2_ release triggered by cation change was not previously obtained.^32^ A following study further investigated the re-speciation reaction in the presence of varied alkali cations and concluded that the cation Lewis acidity sensitively affects the rate and extent of the above reaction. For instance, weaker K^+^ cations undergo negligible interaction with carbamic acid and form negligible amounts of carbamate compared to the stronger Lewis acid Li^+^ cations.^33^ In principle, such changes could be conducted dynamically and potentially reversibly, but such a concept has not, to the best of our knowledge, been developed.

In this work, we demonstrate that such a process can be exploited in an electrochemical configuration designed to actively trigger changes in the metal cation population in an electrolyte and drive cyclical changes in the CO_2_ loading under isothermal (room-temperature) conditions. An electrochemical cation-swing process is described that alternates between dominance of weak or strong Lewis acid cations in the electrolyte. By charging or discharging the cell, the thermodynamically favored amine species are reversibly toggled between carbamic acid or carbamate, allowing CO_2_ to be absorbed or released from the cell, respectively. Factors influencing this conversion process are investigated in detail, including the selection of ionic species (Li^+^, K^+^, Ca^2+^, Mg^2+^, or Zn^2+^) and amine-to-ion concentration ratios, and are supported by detailed solution ^1^H NMR and additional product characterization to validate CO_2_ loading changes in the different ion environments. We further validate performance in a two-electrode electrochemical cell combining an ion-intercalating K^+^ cathode and a Zn metal foil anode, which allows for the ion populations in solution to be cyclically and controllable modulated. These modulations are shown to couple directly with CO_2_ capture and release in the cell, with good agreement between theoretical and observed CO_2_ loading changes. Finally, we examine the preliminary energy requirements and longevity of such a cell and show good stability over 30 cycles.

## Results and Discussion

### Electrolyte Parameter Exploration

**Figure 1:**
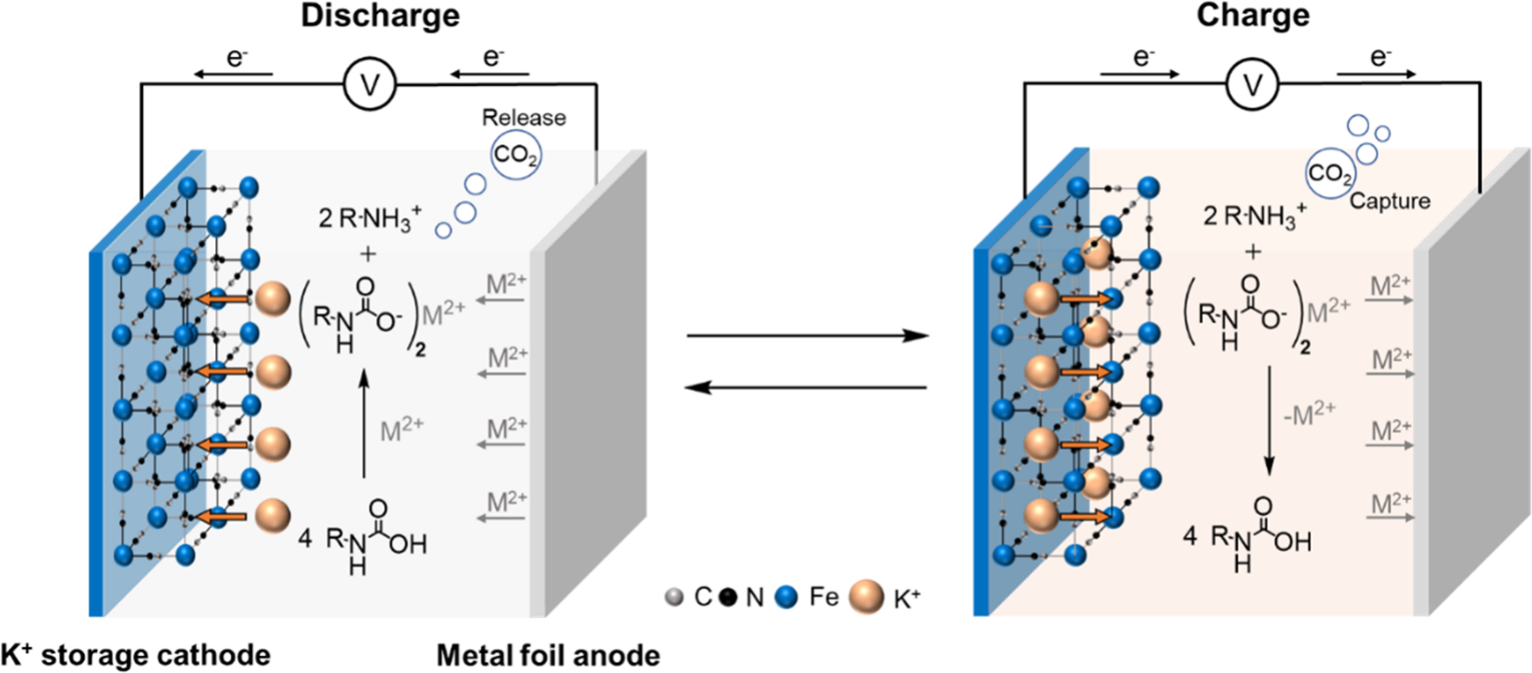
Schematic of a cation-swing CO_2_ separation cycle. Working principle: Discharging the dual-ion cell (left) intercalates K^+^ into the K^+^ storage cathode and strips M^2+^ from the metal anode. M^2+^ then reacts with carbamic acid (RNHCOOH) to form carbamate (RNHCOO^–^) and release CO_2_. Subsequently, charging the cell (right) de-intercalates K^+^ from the K^+^ storage cathode and plates M^2+^ back onto the metal anode, which favors re-formation of carbamic acid and enables additional CO_2_ to be captured by the amine.

Figure 1 shows the general cyclic scheme of the dual-ion cell during discharge and charge. K^+^ is used as the weak Lewis acid cation in the electrolyte given that previous studies have shown it has minimal interaction with carbamic acid and maintains high amine–CO_2_ loading.^33^ In implementing this scheme, a representative K^+^ intercalation cathode with high redox potential (2.5–4.0 V vs K/K^+^, specified in further detail in following text)^34^ was utilized instead of K metal foil to avoid hydrogen evolution upon direct reaction with ammonium cations. A metal foil M, yielding a cation (M^+^ or M^2+^) upon oxidation with Lewis acidity stronger than that of K^+^, serves as the counter electrode.

**Figure 2:**
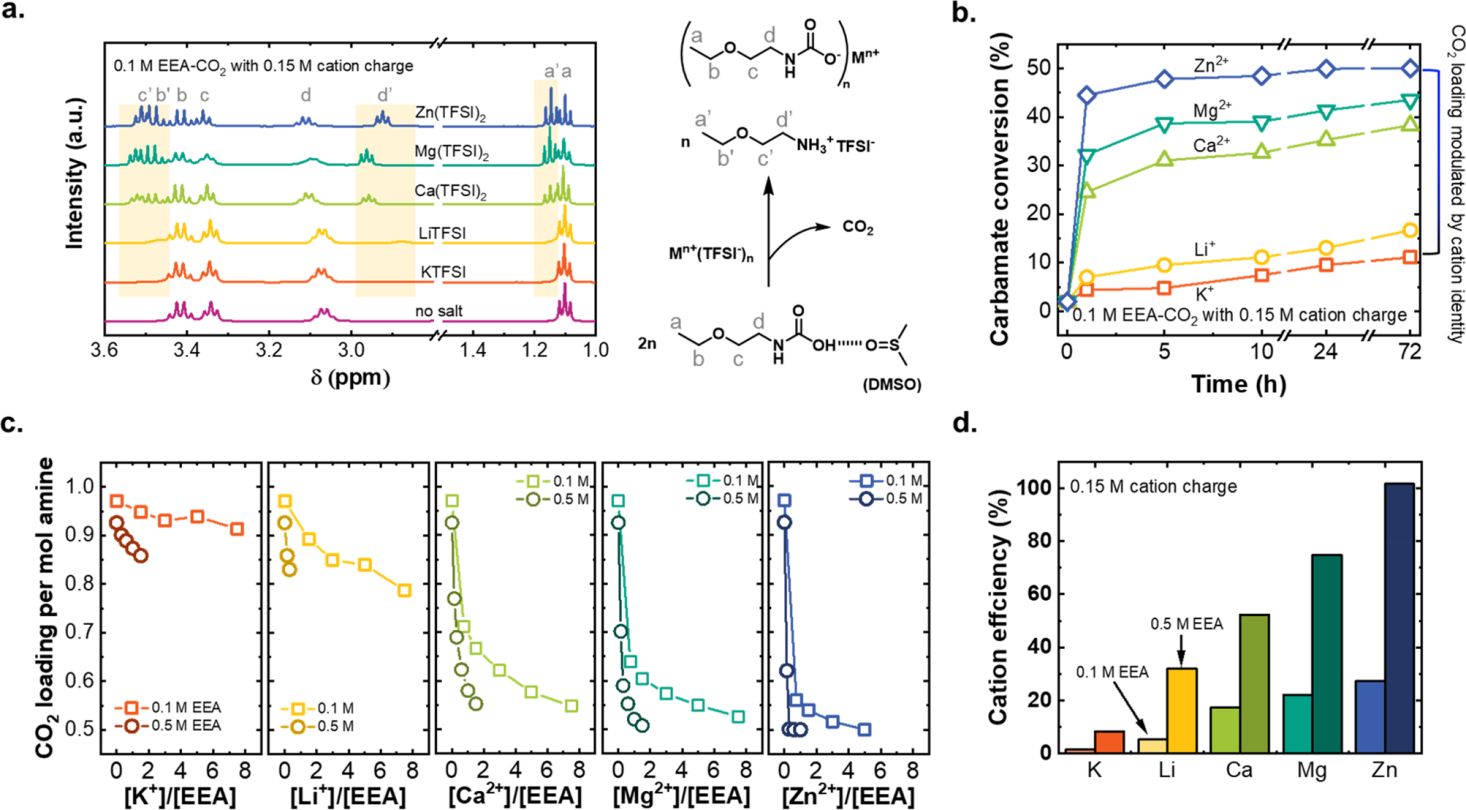
(a) ^1^H NMR of DMSO-*d*_6_ electrolyte containing 0.1 M EEA-CO_2_, taken 24 h after the addition of TFSI-based metal salt as indicated (0.15 M for monovalent cations and 0.075 M for divalent cations). (b) Carbamate conversion determined from (a) as a function of time after salt addition (see Experimental Methods for details). (c) CO_2_ loading of 0.1 and 0.5 M EEA-CO_2_ in DMSO in the presence of different concentrations of cations, taken 2 h after the addition of salt into a saturated EEA-CO_2_ solution in DMSO. (d) Cation efficiency of converting carbamic acid to ammonium carbamate (defined as the molar increase in carbamate after salt addition compared to the injected cation charge concentration in the electrolyte) based on 0.15 M cation charge.

To identify a suitably strong Lewis acid co-cation to achieve a reasonable CO_2_ loading swing, ^1^H NMR was first used to screen the influence of several candidate ions on the amine speciation. Figure 2a shows the resulting spectra of 0.1 M EEA-CO_2_ in DMSO without salt or with 0.15 M cation charge concentration of various cosalts: KTFSI, LiTFSI, Ca(TFSI)_2_, Mg(TFSI)_2_, or Zn(TFSI)_2_ (i.e., 0.15 M for monovalent cations and 0.075 M for divalent cations), where salt was added after the solutions were purged with CO_2_. The TFSI^–^ anion was used in all cases to support a high level of salt dissociation. For the spectra of 0.1 M EEA-CO_2_ without salt, peaks at 1.1, 3.1, 3.3, and 3.4 ppm (labeled (a), (d), (c), and (b)) are from carbamic acid. In contrast, when cosalt was added to solution, additional peaks emerged in all cases at around 1.2, 3.0, 3.4, and 3.5 ppm (a′, d′, b′, and c′) attributed to the formation of ammonium cations.^32^ The formation of ammonium implies an equimolar amount of carbamate (Figure 2a reaction scheme), which shares the same ^1^H NMR peak with carbamic acid due to fast proton exchange and cannot be independently distinguished.^27^ Comparing the peak area ratios of ammonium to that of the carbamic acid for each cosalt enabled quantification of each species in the electrolyte (Figure S1 and Experimental Methods). These results are summarized in Figure 2b, which shows the proportion of carbamate in the presence of each ionic species as a function of the reaction time prior to NMR sample acquisition. Note that the detected equilibrium conversion amounts measured by NMR will depend sensitively on several factors. These include the CO_2_/Ar purge time and gas purge flow rate, which in turn establish the CO_2_ partial pressure and solution concentrations of CO_2_ (Figure S2 and corresponding discussion).

Consistent with prior results, the addition of 0.15 M K^+^ induced little speciation change, reaching only 5% carbamic acid conversion to carbamate after 5 h and stabilizing at 11% after 72 h (compared to a maximum possible change of 50%). Meanwhile, conversion in the presence of Li^+^ was higher, reaching 9.5% and 17% after 5 and 72 h respectively, due to the higher Lewis acidity of the monovalent cation compared to K^+^.^33^ The conversion extent and rates among divalent cations (Ca^2+^, Mg^2+^, and Zn^2+^, 0.075 M) also increased in a manner proportional to Lewis acidity (Ca^2+^ < Mg^2+^ < Zn^2+^)^35,36^ and were yet higher, yielding 25, 32, and 44% in 5 h. Cation-dependent trends are further examined in Figure 2c, which compares the speciation after 2 h but now as a function of the injected salt concentration over a broader range (0.0–0.75 M). For all cations, the CO_2_ loadings decreased monotonically for 0.1 M EEA with increasing cation concentration. This trend was weakest for K^+^ which yielded only minor speciation change, even up to a large salt excess of 0.75 M ([K^+^]/[EEA] = 7.5, 0.92 mol of CO_2_/mol amine). This further supports the assertion that K^+^, the weakest Lewis acid cation, can be added to carbamic acid solutions up to relatively high concentrations without significantly perturbing the carbamic acid population, and thus the CO_2_ loading. Meanwhile, full conversion of 0.1 M EEA to carbamate could be achieved with 0.5 M Zn^2+^, albeit with a larger excess Zn^2+^ concentration than is minimally required to achieve charge balance (0.025 M), i.e. if all Zn^2+^ interacted directly with EEACOO^–^. In other words, dilute amounts of EEA require large excess cation concentrations to drive the conversion reaction to completion. This is reflected in the relatively low cation conversion efficiencies plotted in Figure 2d, which represents the molar increase in carbamate after salt addition compared to the injected cation charge concentration in the electrolyte (cation conversion efficiency = increased carbamate concentration [M]/injected cation charge concentration [M]). Cation conversion efficiency is a measure of the strength of a cation to convert carbamic acid to ammonium carbamate and release CO_2_. This parameter ranges from 0 (no carbamic acid conversion) to 100%, indicating that every charge of the added cations participates in the conversion of carbamic acid to carbamate. Even in the best case of Zn^2+^, the cation conversion efficiency reached ∼20% for 0.1 M EEA.

On the other hand, more extensive and rapid conversion was achieved in all cases by increasing the amine concentration to 0.5 M EEA-CO_2_ (Figure 2c, dark data points). Even without salt, the CO_2_ loading on amines, corresponding to carbamic acid, decreased slightly to 0.93 mol of CO_2_/mol of amine for 0.5 M EEA compared to 0.97 mol of CO_2_/mol of amine for 0.1 M. For K^+^-injected solutions, the CO_2_ loading remained at ∼0.9 mol of CO_2_ per amine even up to 0.5 M of added salt ([K^+^]/[EEA] = 1). In the case of LiTFSI solutions, increasing the total amine and salt concentrations led to noticeable precipitate formation, which upon extraction and analysis by ^7^Li NMR was found to be a Li-carbamate salt (Figure S3), rendering Li^+^ salts nonviable for the more practical amine and salt concentration ranges. No such precipitation was observed in the other cases. As before, the highest conversion extents and rates were achieved with Zn^2+^, which closely approached a 100% cation efficiency with 0.5 M EEA-CO_2_. Overall, these findings imply that the CO_2_ loading change between a 100% K^+^ or a 100% Zn^2+^ solution approaches ∼40%, which is closest to the theoretical maximum of 50% possible in any scenario. All further experiments therefore use K^+^/Zn^2+^ as the dual salt for the cation swing.

### Confirmation and Driving Force for CO_2_ Release

**Figure 3:**
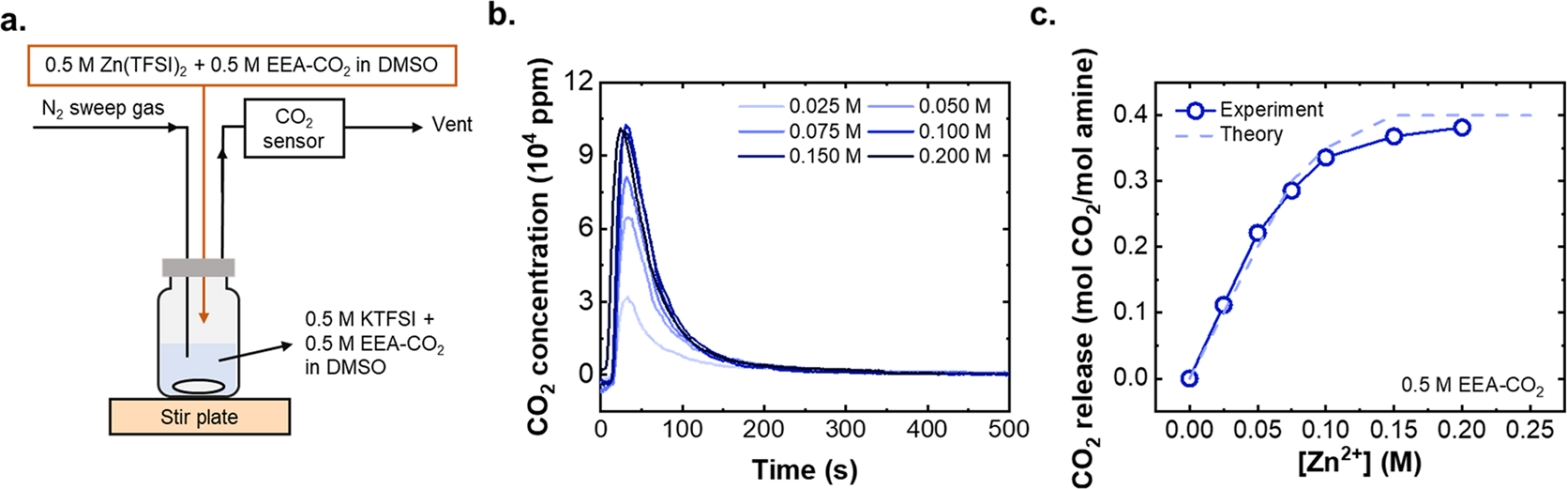
(a) Experimental setup used to chemically validate cation-induced CO_2_ release upon introducing Zn^2+^/amine to K^+^/amine solutions. (b) Real-time CO_2_ concentration (background-corrected for ambient CO_2_, raw data in Figure S4) measured by a CO_2_ sensor after injecting Zn(TFSI)_2_ electrolyte (legends denote the final Zn^2+^ concentration of each curve). (c) Integrated amount of CO_2_ release from Figure 3b at each Zn^2+^ concentration, compared to the expected amount from NMR measurements (Tables S2 and S3 in the Supporting Information).

To directly confirm the release of CO_2_ upon salt injection, an experimental setup was designed to simulate the cation-swing behavior with corresponding gas chromatography analysis (Figure 3a). In this experiment, an electrolyte containing 0.5 M Zn(TFSI)_2_/0.5 M EEA-CO_2_ was injected into an electrolyte containing 0.5 M KTFSI/0.5 M EEA-CO_2_. EEA-CO_2_ was included in both solutions to retain a constant amine concentration, while the injection led to an increase in Zn^2+^ and decrease in K^+^ concentration (see Table S2 and related discussion). This experiment is a better probe of an electrochemical swing process in a realistic device, which will be described later, than the NMR experiments because it can provide direct quantitative evidence of the CO_2_ release. As the final injected Zn^2+^ concentration was further varied from 0 to 0.15 M (Figure 3b, raw data in Figure S4), the measured CO_2_ release increased monotonically and matched quantitatively with the expected amounts based on ^1^H NMR speciation data (Figure 3c, and details in Tables S2 and S3). Beyond [Zn^2+^] = 0.20 M, the released CO_2_ noticeably slowed, indicating that most of the carbamic acid was converted to ammonium carbamate at [Zn^2+^] ≈ 0.15 M. Overall, this experiment provides important support to validate the carbamic acid-to-carbamate reaction mechanism.

**Figure 4:**
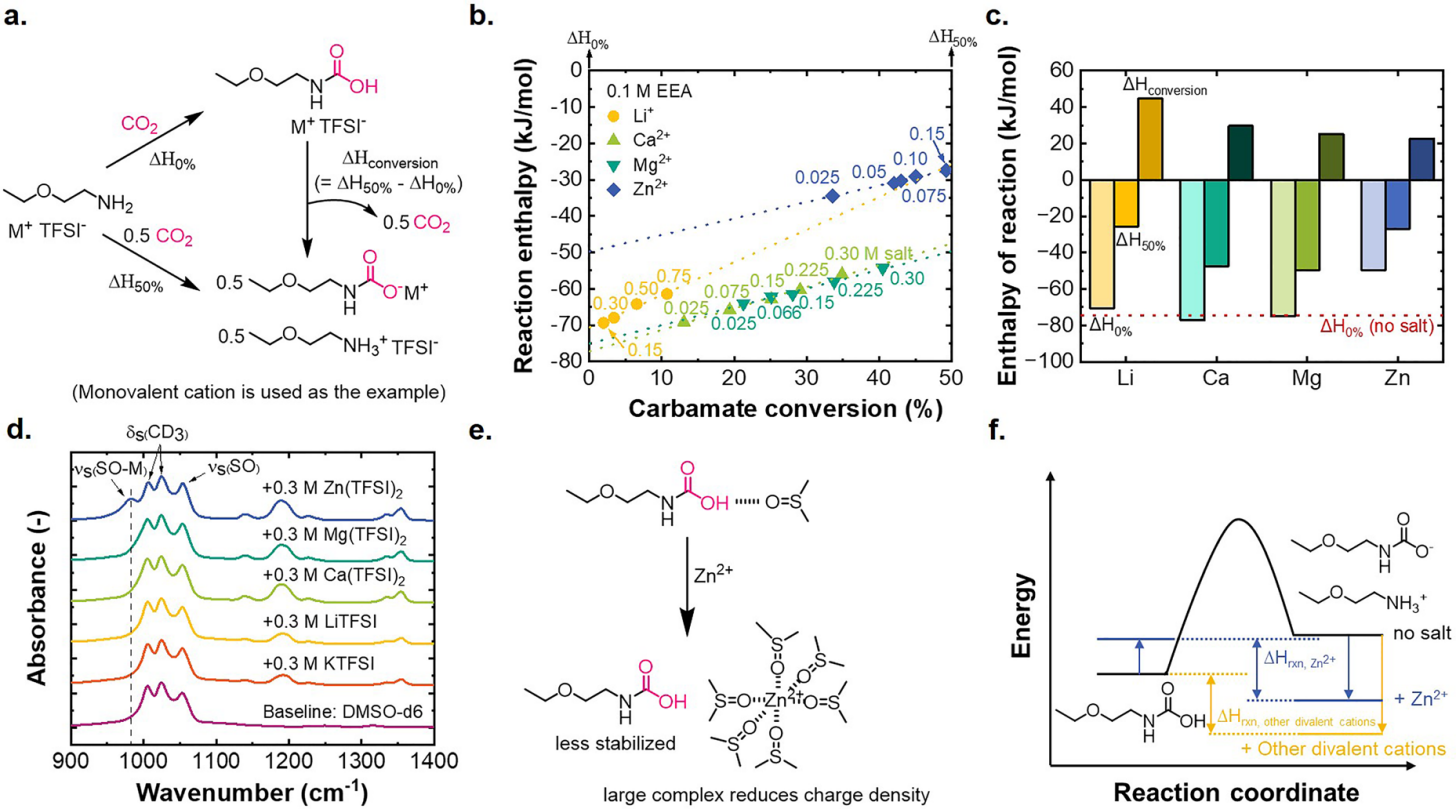
(a) Reaction schemes for carbamic acid (Δ*H*_0%_) or carbamate/ammonium (Δ*H*_50%_) formation from lean amine and CO_2_, and their interconversion (Δ*H*_conversion_). (b) Reaction enthalpy after purging CO_2_ into solution vials containing 0.1 M EEA/DMSO + different concentrations of Li^+^, Ca^2+^, Mg^2+^, or Zn^2+^ cations (labels on each data point denote salt concentration). (c) Comparison of Δ*H*_0%_, Δ*H*_50%_, and Δ*H*_conversion_ in the electrolytes with various cations. (d) FTIR spectra of the DMSO-*d*_6_ electrolyte containing 0.3 M M(TFSI)_*n*_ (M^*n*+^ = K^+^, Li^+^, Ca^2+^, Mg^2+^, and Zn^2+^) (no amine). Note that DMSO-*d*_6_ was used instead of protonated DMSO to prevent potential peak overlap in the region of 900 to 1000 cm^–1^.^38^ (e) Proposed molecular interactions in Zn^2+^-containing electrolyte. (f) Deduced energy landscape of carbamic acid and ammonium carbamate in the examined electrolytes for Zn^2+^ vs. other cations.

The thermodynamic driving force of the carbamic acid-to-carbamate conversion was also experimentally investigated. While EEA-CO_2_ is highly stabilized as carbamic acid in pure DMSO, adding salt may destabilize carbamic acid (reactant) and/or stabilize the ammonium carbamate (product). To better understand which energy level shift dominated this speciation change, gas-flow reaction microcalorimetry (Figure 4) was conducted to measure the enthalpy of the reaction between EEA/CO_2_ in the different electrolyte environments. In the experiments, lean amine (0.1 M) was reacted with CO_2_ under isothermal conditions (*T* = 25 °C) over a range of salt concentrations. For all solutions except pure EEA in DMSO, CO_2_ purging creates a combination of carbamic acid and carbamate (Figure 4a), with the measured enthalpy linearly proportional to the degree of conversion (Figure 4b). The linearity of enthalpy with loading enabled extrapolation to theoretical limits of Δ*H*_0%_ (forming 100% carbamic acid in DMSO with salt) and Δ*H*_50%_ (forming 100% ammonium carbamate in DMSO with salt), both of which cannot be measured directly because such extrema states are never reached in practice with salt present. The difference between Δ*H*_0%_ and Δ*H*_50%_ allows for determination of Δ*H*_conversion_ (= Δ*H*_50%_ – Δ*H*_0%_) (Figure 4a), corresponding to the enthalpy of cation association with carbamic acid and subsequent conversion to carbamate + ammonium + CO_2_.

In all cases, increasing the salt concentration led to less negative enthalpies of reaction. This occurs because fewer total N–C bonds form with a higher salt concentration (carbamic acid: 1 N–C bonds per amine; ammonium carbamate: 0.5 N–C bonds per amine). Li^+^, Ca^2+^, and Mg^2+^ behaved similarly, with Δ*H*_0%_ of −71, −77, and −75 kJ/mol, respectively. These values are essentially the same as those obtained by purging 0.1 M EEA in DMSO with CO_2_ in the absence of any salt (−74 kJ/mol), where slight differences are due to minor amounts of carbamate formation with the salts present. This result indicates that these salts do not significantly perturb the stabilization of carbamic acid by DMSO. Δ*H*_50%_ (stabilization of carbamate) was, however, sensitive to salt and became increasingly negative as the Lewis acidity of the cations increased from Li^+^ (Δ*H*_50%_ = −26 kJ/mol) to Ca^2+^ (−48 kJ/mol) and Mg^2+^ (−50 kJ/mol), as also summarized in Figure 4c. This can be rationalized by the fact that stronger Lewis acid cations have stronger electrostatic interactions with the carbamate anions.^37^

In contrast to the other cations, Zn^2+^ exhibited unique behavior, where both Δ*H*_0%_ (−50 kJ/mol) and Δ*H*_50%_ (−27 kJ/mol) were less negative compared to the other cations. The reason behind the distinct Δ*H*_0%_ can be rationalized by Fourier transform infrared (FTIR) spectra of amine-free electrolytes containing only DMSO and 0.3 M of each salt, which allows for closer interrogation of the cation solvation environments (Figure 4d). Among all cases, only the Zn electrolyte showed a distinct peak at 982 cm^–1^, which has been reported to correspond to the S=O stretching vibration band of metal-DMSO complexes.^38,39^ We therefore propose that the strong Zn-DMSO interactions compete with the hydrogen-bonded stabilization of the carbamic acid and underlie its projected relative destabilization (Figure 4e). Such interactions also screen solvated Zn^2+^ interactions with carbamate, explaining the lower magnitude of Δ*H*_50%_. Overall, Δ*H*_conversion_ was lowest and thus the least endothermic for Zn^2+^ (22 kJ/mol, Figure 4c) compared to Li^+^ (45 kJ/mol), Ca^2+^ (30 kJ/mol), and Mg^2+^ (25 kJ/mol), which aligns well with the rank of cation conversion efficiencies found above. Interestingly, this endothermic yet spontaneous carbamic acid-to-carbamate conversion (in the presence of salt) indicates that the conversion reaction is entropically driven in all cases.

### Electrochemical Cell Design and Testing of Cyclical CO_2_ Capture/Release

Based on the above results, an electrochemical K^+^-Zn^2+^ dual ion cell containing a Prussian White (PW, K_2_Fe^II^[Fe^II^ (CN)_6_]) cathode and Zn anode was conceived to realize the ion swing under electrochemical driving conditions. PW is a well-studied K^+^ intercalation material that attains facile (de)intercalation and cyclability in various electrolytes,^40,41^ and thus was chosen as an exemplar K^+^ storage electrode (characterization in Figure S5). For the anode, Zn metal has been shown to have high and stable Columbic efficiency (CE) of >99% in DMSO-based electrolyte,^42^ supporting the use of Zn foil as the Zn^2+^ storage electrode given that there are only limited choices of’ Zn intercalation materials at present.^40,41^ The electrolyte comprised an initially K^+^-rich solution with minor amounts of Zn^2+^, i.e., 0.5 M KTFSI + 0.1 M Zn(TFSI)_2_, with 0.5 M of EEA that had been prepurged with CO_2_ prior to cell assembly to avoid direct contact between lean amine and Zn metal (the cell atmosphere is also purged with CO_2_ as detailed in the Supporting Information). Note that Zn(TFSI)_2_ is included in the electrolyte since the first cell charging normally requires Zn^2+^ plating from the electrolyte.

**Figure 5:**
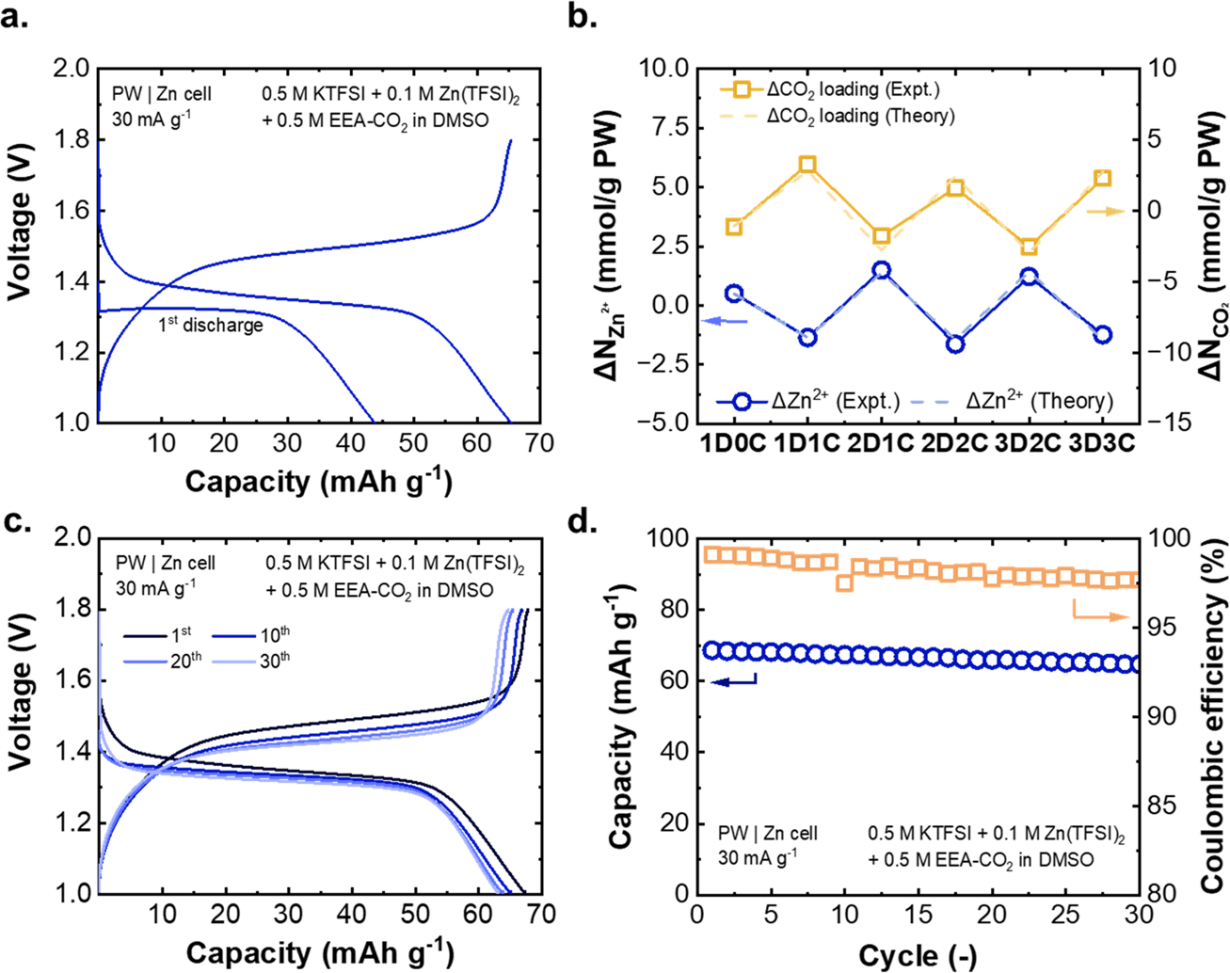
(a) Charge/discharge curves of the PW/Zn full cell at 30 mA g^–1^. (b) Changes of Zn^2+^ concentration and CO_2_ loading on amines at each cell cycling step (C, charge; D, discharge; the delta value of each step is obtained by subtracting the value from the previous step to that of current step). (c) Two-electrode charge and discharge curves for a PW/Zn full cell with amine at 30 mA g^–1^ for 30 cycles. (d) Long-term cycling performances of the PW/Zn full cell at 30 mA g^–1^ for 30 cycles.

PW/Zn full cells were constructed with the electrolyte described above to verify that the cation-swing process examined previously can be driven within an electrochemical cell. A custom two-electrode electrochemical cell outfitted with valves for headspace purging (Figure S6) was used in the following experiments to maintain CO_2_ headspace during cell cycling. Figure 5a shows the galvanostatic charge and discharge curves of the PW/Zn cell at a constant current of 30 mA g^–1^ normalized to the cathode mass. The cell, which was capacity-limited by the PW electrode, demonstrated an initial discharge capacity of 43 mAh g^–1^ and a second discharge capacity of 65 mAh g^–1^. The initial discharge capacity, corresponding to potassiation of PW, is normally not present in stoichiometrically synthesized PW cathodes which are fully reduced, but was accessible here due to partial reversible oxidation of PW during synthesis. The higher capacity corresponds closely to the theoretical capacity of PW with 1 K^+^ intercalation (78 mAh g^–1^) below 1.8 V, given that the second K^+^ intercalation—which corresponds to the full of theoretical capacity 155 mAh g^–1^—occurs at >2.0 V, which exceeds the anodic stability of DMSO.^41,43^

We next validated whether significant changes in the Zn^2+^ concentration could be achieved in the dual-ion cell. To do so, a series of cells was cycled to varying states (1st discharge, 1 full cycle, *etc*.). The electrolyte was then extracted for analysis by inductively coupled plasma–optical emission spectrometry (ICP-OES; see Experimental Methods for details). Given the cell capacity of 65 mAh g^–1^ PW, the expected amount of Zn^2+^ and CO_2_ swing are 2.4 and 4.8 mmol g^–1^ PW between charge and discharge states, assuming each Zn^2+^ modulates an additional 2 CO_2_ release corresponding to 100% cation conversion efficiency as defined in the previous section. Figure 5b confirms a clear linear increase and decrease as the Zn^2+^ concentration was cycled, which matched quantitatively with the theoretical values calculated based on the cell capacity. This result indicates that the cation-swing process worked as expected to modulate the cation population in solution without significant side reactions. The changes of amine CO_2_ loading over discharge/charge was also directly verified by extracting electrolyte from cells at different states, titrating with acid and quantifying the released CO_2_ by gas chromatography (GC, details in the Experimental Methods), and agreed with the expected CO_2_ loading changes based on changes of Zn^2+^. These findings provide the necessary direct evidence that altering the cation populations electrochemically drives changes in the CO_2_ loading in the electrolyte.

Finally, the long-term cycling performance of the PW/Zn cell was evaluated (Figure 5c). With amines and CO_2_ in the cell, 94.2% of the capacity was retained after 30 cycles (Figure 5d), which is only slightly lower than that of cells without amine (96.2%, Figure S10; three-electrode cell measurements in Figures S11–S12). Given that these cells utilize a Zn metal anode which acts as a quasi-infinite reservoir, the observed losses arise at the PW cathode. To further understand the intrinsic Zn cyclability in the two electrolytes, two-electrode Zn/Cu cells were also examined (Figure S13). The Zn plating/stripping CEs of the amine-containing cell were initially lower than without amine but eventually approached and even exceeded that with amine, yielding 96.4% from the 20th to the 50th cycle compared to 95.1% without. A higher degree of Zn plating/stripping polarization was also observed with amine present. The initial CE discrepancy of Zn/Cu cells with and without amine was not due to Zn loss (corrosion) to the solution during cycling, given that the Zn^2+^ concentration in the electrolyte remained constant at 0.10 M after cycling with amines present (Figure S14). We could also confirm that no H_2_ and CO evolution, such as from possible parasitic decomposition of amines, occurred during Zn cycling by extracting the headspace gas from the cell for GC gas quantification (Figure S15). Therefore, the different initial CE values can be attributed to initial electrode conditioning differences in forming the solid electrolyte interface (SEI) on the Cu current collector, which is common for metal plating/stripping reactions. Given the above observations, the small capacity and cycling differences without and with amine in full cells are hypothesized to arise from cell polarization differences arising primarily at the Zn anode, which can lead to capacity slippage of PW in the full cell configuration and will be the subject of focused future work, including development of optimized cycling protocols. Regardless, the good Zn CE and overall PW/Zn cell cycling performance indicate reasonable stability suitable for further development.

Based on the above data obtained under pure (100%) CO_2_ partial pressure, the electrical energy of the cation-swing capture-release process reported herein was calculated to be ∼22–39 kJ/mol CO_2_ at an equivalent areal current of 0.1–0.5 mA cm^–2^ (calculations in the Experimental Methods). This range represents a minimum energy estimate, which will be larger if capture is conducted at lower partial pressures relevant for practical applications. It was estimated that an additional minimum ∼50% increase in electrode capacity and ∼85% increase in energy cost per mol CO_2_ separated would be required to capture CO_2_ from a dilute stream (0.18 bar) and release at 100% purity (atmospheric pressure, 1 bar) for the same amine concentration and voltages used herein. These increases are attributed to the need to overcome the differences in CO_2_ solubilities in DMSO under different partial pressures. On the other hand, increasing the amine concentration and/or decreasing the CO_2_ solubility of the solvent by moving beyond DMSO are effective strategies to limit this additional energy requirement to within ∼20–30% (see discussion in Supporting Information). Overall, the range of energy values obtained and projected in this work are on par with those of other early stage electrochemical processes tested at the laboratory scale (e.g., ∼33 kJ/mol CO_2_ at ∼0.25 mA cm^–2^ for proton concentration process, operating between low and high partial pressures;^25^ and ∼56 kJ/mol CO_2_ at ∼0.5 mA cm^–2^ for quinone chemistry tested under pure CO_2_ inlet conditions;^21^ the latter quinone systems have seen more extensive development).

Currently, the performance of the cation swing process is limited by the capacity and rate of the PW cathode, which modulates the total amount of CO_2_ loading change and rate of capture/release for a given electrolyte volume. Looking ahead, low-capacity materials are not ideal for practical application given that they will require significant oversizing in real cells to achieve realistic capacity changes in the electrolyte. Therefore, future designs of the cation-swing process should seek to move beyond the use of PW cathodes to enhance overall capacity and stability, which may involve consideration of other ion intercalation materials, metal alloys, or metals. Beyond the cathode limitations, the electrolyte components in the cation-swing process provide significant opportunity for further exploration given that the amine, salt anion, and solvents chosen in this work are not extensively optimized. The role of the amine structure is expected to be of particular significance given differences in CO_2_ binding strengths, which are anticipated to affect the relative stability and speciation of amine/ion interactions, capture/release kinetics, and secondary effects such as solution viscosity and transport limitations, which were not investigated in detail here. Other nonaqueous solvents having different hydrogen bonding strengths with amine-CO_2_ adducts would also be of interest to finely tune the stability of the carbamic acid and the solvation of Lewis acid cations. Finally, although the cation swing process currently resembles a batch reactor (discrete states for CO_2_ capture and release), a continuous cation swing process, which aligns well with current CO_2_-emitting industrial processes, is a possible next step with suitable design of the cell architecture and will be in the scope of a future study.

## Conclusions

An electrochemical cation-swing process is described for CO_2_ separation as a possible alternative to thermal regeneration, as used today with most amine scrubbing processes. The proof-of-concept system described in this first study demonstrates the ability to reversibly modulate the CO_2_ loading on an amine under ambient conditions by electrochemically swinging the cation concentrations in the electrolyte between Zn^2+^ (discharge) and K^+^ (charge). The underlying principle of this process was shown to exploit a carbamic acid-to-carbamate conversion process driven by Zn^2+^ cations, where the role of Zn^2+^ in this system is to (1) destabilize carbamic acid by reducing the overall hydrogen bond strength from DMSO and (2) stabilize carbamate by electrostatic interactions. Without extensive optimization, the current process revealed a minimum energy requirement of ∼22–39 kJ/mol of CO_2_ at ∼0.1–0.5 mA cm^–2^ for capture/release between pure (100%) CO_2_. At the cell level, the possibility to extend this concept to a wider landscape of amines, solvents, electrodes, and cell designs presents an opportunity for further significant process improvement. Looking ahead, better understanding of the factors governing the kinetics of the carbamic acid-to-carbamate reaction, as well as long-duration stability upon cycling, is critical to enable modeling efforts to evaluate and advance the upper-bound performance of this process.

## Supporting Information Available

Experimental methods, ^1^H NMR spectra of 0.1 M EEA-CO_2_ with no salt in DMSO, carbamate conversion with different sample preparation procedure, ^7^Li NMR spectra of the lithium carbamate precipitate, raw data of CO_2_ release after salt injection, XRD and SEM of synthesized PW materials, design of the custom two-electrode electrochemical cell, ICP calibration curve for Zn^2+^, GC calibration curve for CO_2_, and additional electrochemical cell data without and with amines (PDF)
Transparent Peer Review report available (PDF)
