# Supplementary material for: Dual Salt Cation-Swing Process for Electrochemical CO2 Separation
Source: ACS Cent Sci. 2023 Aug 30;9(9):1750–7. doi: 10.1021/acscentsci.3c00692 (PMC10540289; doi:10.1021/acscentsci.3c00692)
Supplement: Supplementary file 1 — oc3c00692_si_001.pdf [file oc3c00692_si_001.pdf]

Dual Salt Cation-Swing Process for Electrochemical CO_2 Separation

Fang-Yu Kuo[a], Sung Eun Jerng[b], Betar Gallant[b].*

[a] Department of Chemical Engineering, Massachusetts Institute of Technology, Cambridge, Massachusetts 02139, United States

[b] Department of Mechanical Engineering, Massachusetts Institute of Technology, Cambridge, Massachusetts 02139, United States

* Corresponding author: bgallant@mit.edu

Supporting Information

Experimental Methods

Safety Statement:

No unexpected or unusually high safety hazards were encountered.

Chemicals and Materials:

Zinc foil (99.9% polycrystalline metallic foil, MTI) and 2-ethoxyethylamine (98%, TCI America) were used as received and stored in the glovebox (H_2O content <1 ppm, O_2 content <1 ppm, MBRAUN). KTFSI (99.5%, Solvionic), LiTFSI (99.99% trace metals basis, Sigma-Aldrich), $Ca(TFSI)_2$ (99.5%, Solvionic), $Mg(TFSI)_2$ (99.5%, Solvionic), $Zn(TFSI)_2$ (99.5%, Solvionic), and Whatman filter paper (grade GF/F, Sigma Aldrich) were dried in a Buchi glass oven under active vacuum overnight at 120 °C before transfer to the glovebox. 4Å molecular sieves (bead size 8-12 mesh, Sigma-Aldrich) were activated in the Buchi glass oven under active vacuum for 24 hours at 250 °C. DMSO (anhydrous, > 99.9%, Sigma-Aldrich) and DMSO-d_6 (99.9 atom % D, Sigma-Aldrich) were dried with 20 vol% activated 4Å molecular sieves for two days before use. All amine, solvents, and salts were stored inside the glovebox at room temperature.

^{1}H Nuclear Magnetic Resonance (NMR) Spectroscopy:

^{1}H NMR measurements were performed using a three-channel Bruker Avance Neo spectrometer (500 MHz), equipped with a 5 mm liquid-nitrogen cooled Prodigy broad band observe (BBO) cryoprobe. All samples were prepared with DMSO-d_6 as the deuterated solvent inside the glovebox and loaded in a capped Wilmad NMR tube (700 µl).

Reaction Microcalorimetry Experiments and Analysis:

Experiments were performed using a Micro Reaction Calorimeter (uRC™, Thermal Hazard Technology) equipped with a gas flow option. In a typical experiment, 0.7 ml of electrolyte comprising 0.1 M EEA and the desired concentration of salt in DMSO-d_6 was loaded into a 1.5 ml stainless-steel vial, which is connected with two 1/16" tubes as the inlet (with an inline flow controller to purge CO_2) and outlet (vent). After loading the vial into the calorimeter, the vial equilibrates until the temperature converges to 25.000°C and signal fluctuation is within 0.01 mW. Subsequently, CO_2 is purged at a flow rate of 1 sccm controlled by the flow controller, and the resulting signal is collected until the heat returns to baseline, indicating saturation. The integrated peak gives the enthalpy of reaction. The speciation of EEA-CO_2 adducts are measured by ^{1}H NMR immediately following the calorimetry experiments. A series of enthalpy vs. carbamate conversion using the same salt allows for fitting of a line through the data points, and the intercept at projected carbamate conversions of 0% and 50% were $\Delta H_{0\%}$ and $\Delta H_{50\%}$.

Fourier-Transform Infrared Spectroscopy (FTIR):

Desired liquid sample is used for FTIR measurements on a Nicolet iS50 FT-IR spectrometer (Thermo Scientific). All measurements were performed in the transmission mode over a wavenumber range of 400 to 4000 cm^{-1} by ATR using a diamond crystal.

Prussian White (PW) Synthesis and Electrode Preparation:

The procedure followed a previously reported method.[1] Briefly, 1.67 g (6 mmol) of iron sulfate pentahydrate and 25 g of potassium citrate were dissolved in a 500 ml beaker with 100 ml deionized water

while N_2 was bubbled at a flow rate of 100 sccm to remove the dissolve O_2. 1.689 g (4 mmol) of potassium hexacyanoferrate(II) trihydrate was dissolved in another beaker with 100 ml deionized water, and was slowly added into the iron sulfate solution while stirring. The mixture was stirred at room temperature overnight with continuous N_2 bubbling. Next, the precipitate was collected by centrifuge and washed with DI water to remove the unreacted reactants. This process was repeated three times to ensure no residual impurities. Finally, the clean product was dried under active vacuum at 100°C overnight. To prepare the PW cathodes, a slurry with 70 wt% PW, 20 wt% Super P, and 10 wt% polyvinylidene fluoride (PVDF) were mixed in N-Methylpyrrolidone (NMP) and then coated on Toray paper (060, wet-proofed, Fuel Cell store) using a doctor blade technique. The obtained PW-coated Toray paper was punched into circular disks (15 mm diameter) and then dried in a Buchi glass oven under vacuum at 100°C overnight. Typical active material loadings were 2.5–4.0 mg/cm^2.

Cell Assembly and Electrochemical Evaluation:

Full-cell performance measurement: Custom two-electrode electrochemical cells were outfitted with valves to enable headspace sampling (Figure S6). Cells consisted of a 15 mm PW electrode, two 18 mm Whatman glass fiber separators, 250 µL of electrolyte, a 15 mm Zn electrode, a stainless-steel (316) mesh, and a spring. The electrolyte was 0.5 M KTFSI, 0.1 M $Zn(TFSI)_2$, and 0.0 M / 0.5 M EEA-CO_2 in DMSO (electrolyte with amine is purged with CO_2 prior to being loaded into the cell). Assembled cells were rested for 6 h before testing. Galvanostatic cycling was performed at 30 mA g^{-1} from 1.0 V – 1.8 V. All electrochemical experiments were conducted on BioLogic or Neware Battery Tester channels.

PW half-cell performance measurement: A glass three-electrode cell (Pine Research, Low Volume Cell) was used in this measurement. The PW working electrode was prepared as described above, and two pieces of Zn foil were used as reference and counter electrodes, respectively. Cells were rested for 6 h before testing. The OCV after 6 h resting is approximately 1.4 V vs Zn/Zn^{2+}. Galvanostatic charge and discharge was performed at 30 mA g^{-1} from 1.05 V – 1.70 V vs Zn/Zn^{2+}. All electrochemical experiments were conducted on BioLogic channels.

Zn anode reversibility test: Custom two-electrode electrochemical cells were outfitted with valves to enable headspace sampling (Figure S6). Cells consisted of a 15 mm Cu electrode, two 18 mm Whatman glass fiber separators, 250 µL of electrolyte, a 15 mm Zn electrode, a stainless-steel (316) mesh, and a spring. Assembled cells were rested for 6 h before testing. Cells with EEA in the electrolyte were purged with CO_2 to maintain a CO_2 headspace before resting. Galvanostatic cycling experiments were conducted at 0.25 mA/cm^2 for 1 h (capacity of 0.25 mAh/cm^2), with a charging (stripping) cutoff voltage of 1 V. After each half cycle, the cell rested at OCV for 5 minutes. Electrochemical experiments were conducted using BioLogic or Neware Battery Tester channels.

Inductively Coupled Plasma - Optical Emission Spectrometry (ICP-OES) Measurements:

ICP-OES experiments were performed in an Agilent 5100 VDV spectrometer with an argon plasma in radial viewing mode. A zinc standard (1000 mg/L Zn in nitric acid, Sigma-Aldrich) for ICP was used to generate the calibration curves based on the observed signal at 334.5 nm.

Gas Chromatography (GC):

Gas samples were extracted with a gas-tight syringe (SGE Gas Tight Syringes, Luer Lock) equipped with a push button to prevent gas contamination, then injected into Agilent 7890B gas chromatograph equipped with a TCD detector and an FID detector with a methanizer.

Calculation of Carbamic Acid-to-Carbamate Conversion Extent by ^{1}H NMR Spectra (Figure 2):

Desired amounts of EEA and electrolyte salt were dissolved in DMSO-d_6, and then the as-prepared electrolyte was purged with CO_2 to form EEA-CO_2 adducts. Then, Ar is used to purge the vial headspace briefly before opening the vial and transferring 700 µL of the electrolyte to a NMR tube inside the glovebox at the indicated sampling time. In a typical ^{1}H NMR spectra (Figure S1), there were two well-separated peaks at high chemical shift which could be used for quantifying the proportion of carbamic acid, carbamate, and ammonium.[2–4] The peak located around 6 ppm is the proton peak of -N**H**-C- from both carbamic acid and carbamate. The other peak located between 7 and 10 ppm, depending on the electrolyte salt, is the proton peak of -COO**H** from carbamic acid in fast exchange with the -N**H**$_3^+$ peak.

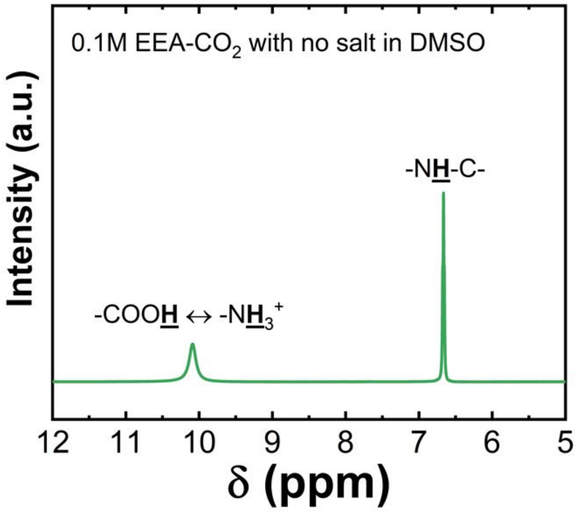

Figure S1. ^{1}H NMR spectra of 0.1 M EEA-CO_2 with no salt in DMSO.

Figure S1 Discussion

We assume x and x' to be the number of amide protons in carbamic acid (R-N**H**COOH) and carbamic acid protons (R-NHCOO**H**), respectively. Then, we assume y and y' to be the number of amide protons in carbamate (R-N**H**COO$^-$) and the protons in the ammonium cation (R-N**H**$_3^+$):

x: R-N**H**COOH

x': R-NHCOO**H**

y: R-N**H**COO$^-$

y': R- N**H**$_3^+$

With these assumptions, we can write down the equation for the total amount of protons in peaks at 6 ppm and 7–10 ppm, respectively. For demonstration, we assume the number of protons at 6 and 7–10 ppm are a and b respectively:

$$x + y = \mathrm{a} \tag{S1}$$

$$x' + y' = \mathrm{b} \tag{S2}$$

In addition to the above equations, we also know that (1) the amount of amide proton in carbamic acid (R-N**H**COOH) equals the number of carbamic acid protons (R-NHCOO**H**) and (2) the molar quantities of ammonium and carbamate are the same in the electrolyte:

$$x = x' \quad \text{(S3)}$$

$$y' = 3y \quad \text{(S4)}$$

The latter follows because of the three-fold higher number of protons in y' than x'. By solving the above equations, one obtains $x = x' = \frac{3a-b}{2}$, and $y = \frac{b-a}{2}$. Here, we define the carbamate conversion by the equilibrium proportion of carbamate in the electrolyte ($\frac{y}{x+y+y'/3}$), where 50% represents a full conversion of this reaction. With this definition, we can obtain carbamate conversion as $\frac{b-a}{a+b}$ ($= \frac{b/a-1}{1+b/a}$). Additionally, the total CO_2 loading is defined as the ratio of carbamate + carbamic acid to the total amine concentration ($\frac{x+y}{x+y+y'/3}$). Table S1 tabulates ratios of peak area at 10 ppm to that at 6 ppm ($\frac{b}{a}$) and their corresponding carbamate conversions and CO_2 loading.

Table S1. Carbamate conversion and CO_2 loading on amine as a function of ratio of peak area at 10 ppm to that at 6 ppm.

Peak area ratio ($\frac{b}{a}$)	Carbamate conversion (%)	CO_2 loading (CO_2 per amine)
1	0.0	1.00
1.25	11	0.89
1.5	20	0.80
1.75	27	0.73
2	33	0.67
2.25	38	0.62
2.5	43	0.57
2.75	47	0.53
3	50	0.50

Effect of Different Sample Preparation Procedures on Carbamate Conversion:

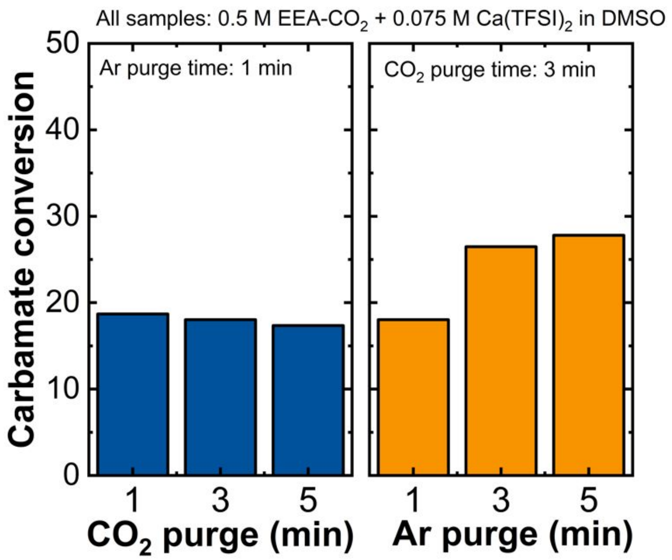

Figure S2. Carbamate conversion measured by ^{1}H NMR (using Ca(TFSI)$_2$ as the model system) with different CO_2 or Ar gas purge times (fixed flow rate at 100 sccm).

<u>Figure S2 Discussion</u>

For a standard ^{1}H NMR sample preparation, CO_2 is bubbled into the electrolyte containing the desired amounts of amine and salt in a gas-tight vial to form amine-CO_2 adducts. Subsequently, the vial headspace was purged briefly with Ar to remove headspace CO_2 prior to opening the vial for electrolyte transfer into an NMR tube, which can otherwise contaminate the glovebox. The subsequently measured carbamate conversion by ^{1}H NMR was noticeably affected by the sample preparation procedure, especially Ar purge time and Ar purge rate, even for controlled amine and salt concentrations in the electrolyte. Different CO_2 purge times were first examined, and the results indicated little effect on carbamate conversion provided enough CO_2 was purged to saturate the electrolyte (typically only ~1 min. needed, Figure S2 left). However, longer Ar purge times increased carbamate conversion significantly (Figure S2 right). This is because purging Ar removes physically dissolved CO_2 in the electrolyte, which is at relatively high concentration in nonaqueous solvent (*e.g.* 0.14 M for DMSO),[5] thus shifting the below equilibrium from carbamic acid to increased ammonium carbamate and decreasing the measured total CO_2 loading on amine. Note that the same effect is observed if Ar is purged with faster flow rates.

$$2\ \text{R-NHCOOH} \leftrightarrow \text{RNHCOO}^- + \text{RNH}_3^+ + CO_2 \quad \text{(S4)}$$

If the electrolyte is not saturated with CO_2 before injecting cations due to long/strong Ar purge in ^{1}H NMR sample preparation, part of the carbamate conversion would be attributed to the equilibrium shift as discussed above instead of cation-induced conversion. This would lead to the overestimation of the cation efficiencies. Therefore, all the Ar purging times (1 min) and rates (100 sccm) are minimized in this work to prevent this equilibrium shift.

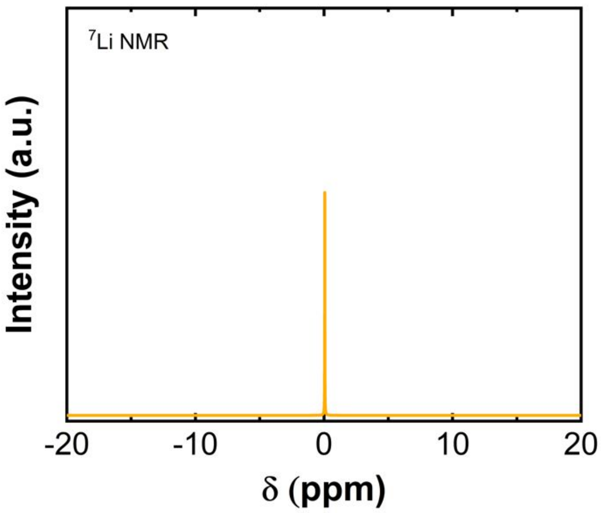

Figure S3. ^{7}Li NMR of the precipitate from 0.5 M EEA/0.50 M LiTFSI in DMSO.

Calculation of the Total Amount of CO_2 Released Due to Electrolyte Injection into Saturated Carbamic Acid Solutions (Figure 3):

In a typical experiment, an electrolyte containing 0.5 M KTFSI/0.5 M EEA in DMSO was loaded in a gas-tight vial (SureSTART™ 10 mL glass screw top headspace vials). A needle/tubing apparatus connected to upstream CO_2 and N_2 gas flow controllers (Alicat Scientific) was plugged through the septum into the electrolyte. A second needle/tubing apparatus provided gas outflow to a CO_2 sensor (SprintIR®-W 100% CO_2 sensor). CO_2 gas flowed at 100 sccm to purge the amine electrolyte with CO_2 for 5 mins (CO_2 loading: 0.91 CO_2 per EEA). Then, N_2 gas flowed in at 200 sccm while an electrolyte containing 0.5 M $Zn(TFSI)_2$/0.5 M EEA-CO_2 in DMSO was injected by syringe into the vial. The same experiment was repeated by injecting electrolyte with 0.5 M KTFSI and 0.5 M EEA-CO_2 in DMSO (*i.e.*, no $Zn(TFSI)_2$) to get the baseline CO_2 change due to injection of the same solution. Note that since the initial electrolyte is purged with CO_2, the baseline headspace CO_2 concentration is high and gradually reduced while purging N_2 during the experiment. This experiment was then repeated for different amount of injected $Zn(TFSI)_2$ (Figure S4). The initial and final concentrations of KTFSI and $Zn(TFSI)_2$ in each case are listed in Table S2, with the total final electrolyte volume was fixed at 5 mL.

Table S2. The amounts of initial 0.5 M KTFSI + 0.5 M EEA-CO_2 in DMSO (KTFSI electrolyte) in the vial, the amounts of injected 0.5 M $Zn(TFSI)_2$ + 0.5 M EEA-CO_2 in DMSO ($Zn(TFSI)_2$ electrolyte), and their corresponding final K^+ and Zn^{2+} concentration. Total amine concentration and electrolyte volume were fixed at 0.5 M and 5 mL, respectively.

Initial KTFSI electrolyte (mL)	Injected $Zn(TFSI)_2$ electrolyte (mL)	Final K^+ concentration (M)	Final Zn^{2+} concentration (M)	Total cation concentration (M)	Total amine concentration (M)	Zn/amine molar ratio
4.75	0.25	0.475	0.025	0.50	0.50	0.05
4.50	0.50	0.450	0.050	0.50	0.50	0.10
4.25	0.75	0.425	0.075	0.50	0.50	0.15
4.00	1.00	0.400	0.100	0.50	0.50	0.20
3.50	1.50	0.350	0.150	0.50	0.50	0.30
3.00	2.00	0.300	0.200	0.50	0.50	0.40

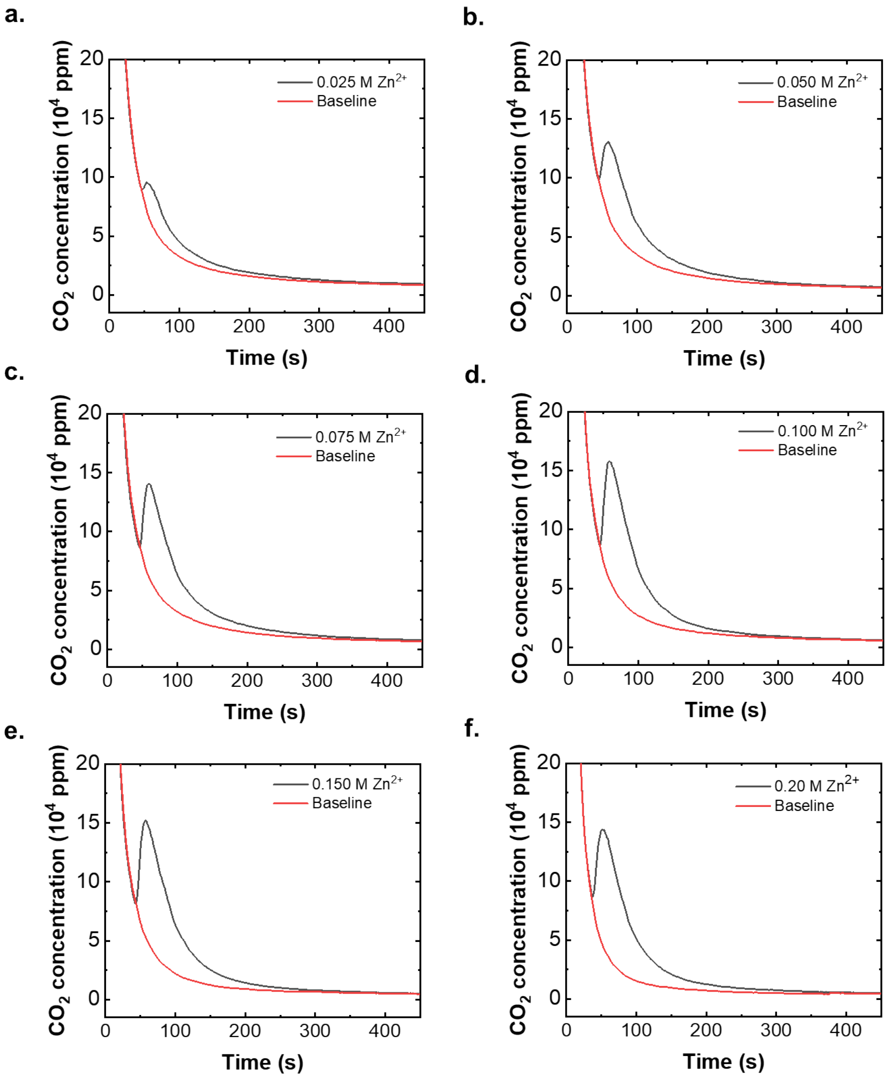

Figure S4. Raw data of CO_2 concentration after (a) 0.025 M, (b) 0.050 M, (c) 0.075 M, (d) 0.100 M, (e) 0.150 M, and (f) 0.200 M $Zn(TFSI)_2$ injection following the methodology described above. Red traces indicate the corresponding baseline in which the high CO_2 concentration in the start arises from the initial CO_2 purge to the electrolyte.

Figure S4 Discussion

After obtaining the peak area of each condition, the amount of CO_2 released per mol of EEA is calculated by the following formula:

$$x_{CO_2} = \frac{P\dot{V}_{N_2}A_{peak}}{RTn_{EEA}} \tag{S5}$$

where x_{CO_2} is the mol of CO_2 release per mol EEA, P is the pressure reading on the flow controller, $\dot{V}_{N_2}$ is the total volumetric flow rate of gas set by the mass flow controller (200 sccm), A_{peak} is the integrated peak area from the experimental data, R is the ideal gas constant, T is the temperature, and n_{EEA} is the total amount of EEA in the final electrolyte (2.5 mmol). The expected mol of CO_2 release per mol EEA is determined as follows. (1) CO_2 loading on amines with the same set of K^+ and Zn^{2+} concentrations was quantified with

^1H NMR, as shown in Table S2. (2) The difference between the CO_2 loading with a given Zn^{2+} concentration and that of 0.00 M Zn^{2+} concentration (0.91) indicated an expected CO_2 release, which is plotted as the dashed line in Figure 3c. This calculation assumes that, despite the presence of K^+ in solution, the speciation is determined entirely by Zn^{2+} concentration. Note that the CO_2 loading for 0.025 and 0.075 M Zn^{2+} are determined by interpolating the CO_2 loading from samples with adjacent concentrations from the NMR data.

Table S3. CO_2 loading of 0.5 M EEA-CO_2 in DMSO (quantified by ^1H NMR) in the presence of different concentrations of K^+ and Zn^{2+} (total cation concentration: 0.5 M). This set of data simulated cation swing between K^+ and Zn^{2+} and obtained the corresponding CO_2 loading on amines, shown as the dashed line (Theory) in Figure 3c.

Final K^+ concentration (M)	Zn^{2+} concentration (M)	CO_2 loading (mol CO_2/mol amine)
0.50	0.00	0.91
0.45	0.05	0.70
0.40	0.10	0.53
0.35	0.15	0.50
0.30	0.20	0.50
0.25	0.25	0.50

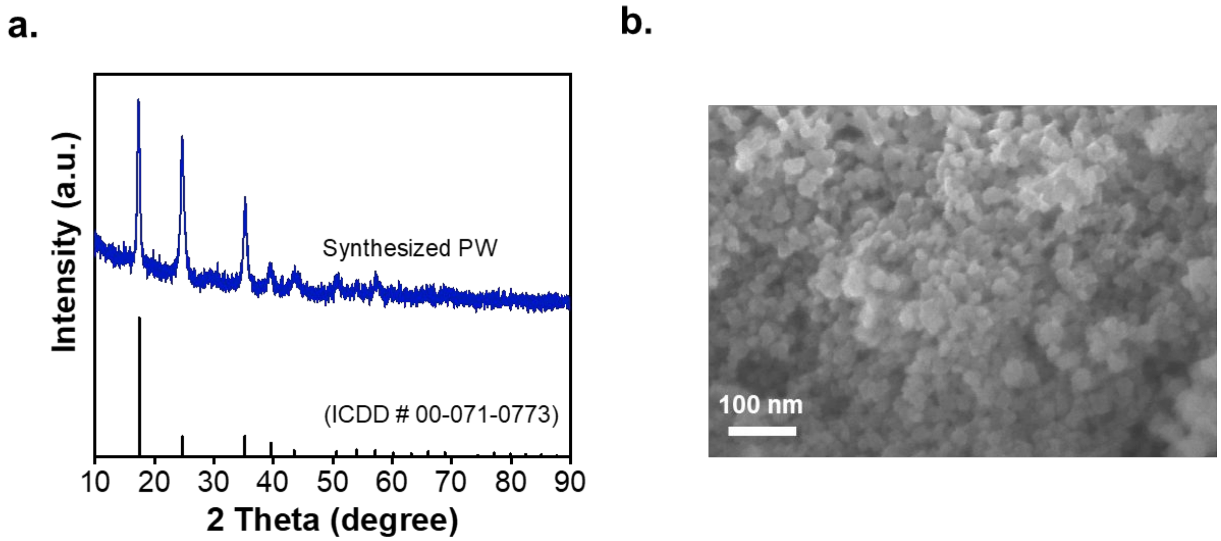

Figure S5. (a) XRD pattern of the synthesized Prussian white (PW). (b) SEM image of the PW particles with the particle size of 30 – 50 nm.

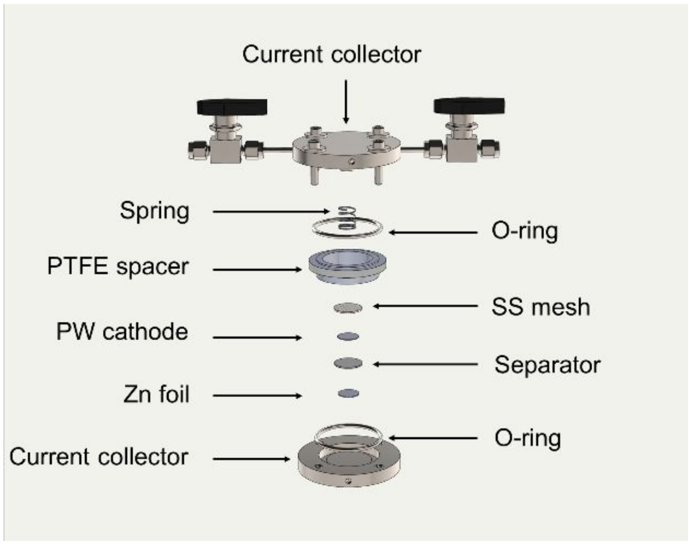

Figure S6. Custom two-electrode electrochemical cells outfitted with valves for headspace purging or sampling.

Inductively Coupled Plasma - Optical Emission Spectrometry (ICP-OES) Measurements:

To prepare ICP samples for PW|Zn cells, the cell was opened after cycling to a specific state and the PW cathode, separator, and Zn foil were soaked in DMSO for 3 h. Subsequently, the DMSO solution was filtered with a syringe filter (PTFE with 0.22 µm pore size, VWR) to remove any solids, then the filtrate was mixed with 3 wt% HNO_3 solution to a final composition of 10 vol% of DMSO and 90 vol% of 3 wt% HNO_3 solution. For analysis of Coulombic inefficiencies of Zn|Cu cells with 0.5 M EEA-CO_2 in the initial cycles, the cells were opened after 9 cycles (conditions: current 0.25 mA/cm^2, capacity 0.25 mAh/cm^2, and 9 plating + stripping cycles). The separators were soaked in 1 ml DMSO for 3 h, and the solution was then filtered with a syringe filter to remove any solids. The filtrate was mixed with 3 wt% HNO_3 solution to a final composition of 10 vol% of DMSO and 90 vol% of 3 wt% HNO_3 solution as the ICP sample for quantification of final Zn^{2+} concentration in the electrolyte. ICP-OES experiments were performed in an Agilent 5100 VDV spectrometer with an argon plasma in radial viewing mode. A zinc standard (1000 mg/L Zn in nitric acid, Sigma-Aldrich) for ICP was used to generate the calibration curves based on the observed signal at 334.5 nm.

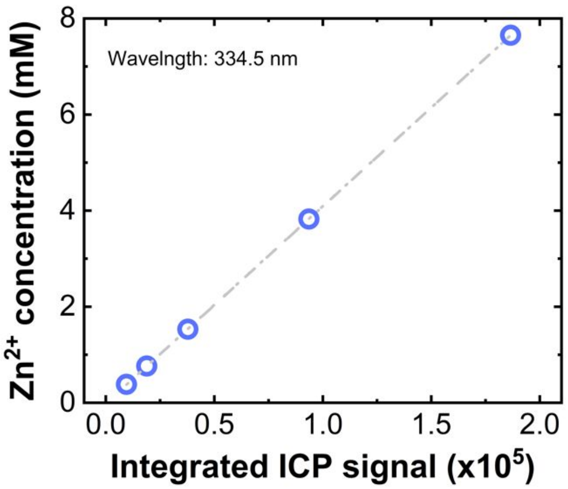

Figure S7. Calibration curve of the Zn^{2+} signal using commercial Zn standard (1000 mg/L Zn in nitric acid).

The theoretical change of Zn^{2+} concentration ($\Delta C_{Zn^{2+}}$) in the electrolyte at each step of cell charge or discharge was calculated as follows:

$$\Delta C_{Zn^{2+}} = \frac{-\Delta Q}{nFV_{electrolyte}} \quad \text{(S6)}$$

Where ΔQ is the charge passed into the Zn anode compared to previous step (C) , n is the cation charge (n = 2 for Zn^{2+}), F is the Faraday constant (96485 C/mol), and $V_{electrolyte}$ is the electrolyte volume in the cell (= 250 µL). Since the material loadings on PW cathode can vary, $\Delta C_{Zn^{2+}}$ is then normalized by PW loading for comparison in Figure 5b.

Quantification of CO_2 Loading on Amine by Gas Chromatography (GC):

Full cells were charged or discharged to a specific point, then disassembled under air atmosphere. Subsequently, 5 ul of the electrolyte was squeezed out from the separator and put into a gas-tight vial (SureSTART™ 10 mL Glass Screw Top Headspace Vials, Thermo Fisher Scientific). Then, excess

amounts of 37 wt% HCl acid (0.2 ml) were injected into the vial to stimulate the CO_2 release from the amine. A sample of the vial headspace was extracted by a gas tight syringe (Luer Lock, Sigma) with a push-button syringe valve (Sigma) 20 minutes after the acid injection to allow the headspace to reach equilibrium. The gas in the syringe was then injected to an Agilent 7890B chromatograph with a flame ionization detector (FID) and a thermal conductivity detector (TCD). The CO_2 signal showed up ~6.7 mins after the injection and the calibration of the TCD detectors for CO_2 were performed by using $NaHCO_3$ as the external standard. Briefly, standard solutions of 5, 12.5, 25, 37.5, and 50 mM of $NaHCO_3$ in H_2O were prepared and 50 µL of each standard solution were sealed in gas-tight vials and injected with 0.2 ml 37wt% HCl. Upon reaction with acid, $NaHCO_3$ releases CO_2 stoichiometrically ($NaHCO_3 + H^+ \rightarrow Na^+ + H_2O + CO_2$), thus allowing a linear calibration of the CO_2 TCD signals with the known amount of CO_2 in the vial headspace (Figure S8). Note that the calibration line does not pass through the origin since the samples are made under atmosphere. Therefore, a background CO_2 concentration (~450 ppm) was recorded.

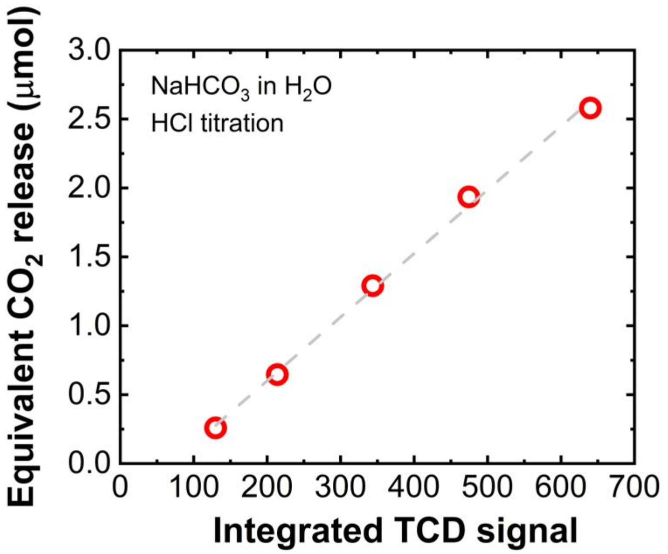

Figure S8. Calibration of the CO_2 signal using $NaHCO_3$ standards.

To get the experimental change of CO_2 loaded on amine (ΔN_{CO_2}), the obtained CO_2 release for each step was compared to its previous step. To calculate the theoretical CO_2 change, the change of Zn^{2+} concentration upon a given discharge or charge step was calculated based on the cell capacity assuming that every Zn^{2+} induces 2 CO_2 molecules to be released (4 RNHCOOH + $Zn^{2+} \rightarrow$ 2 ($RNHCOO^-$)Zn^{2+} + RNH_3^+ + 2 CO_2).

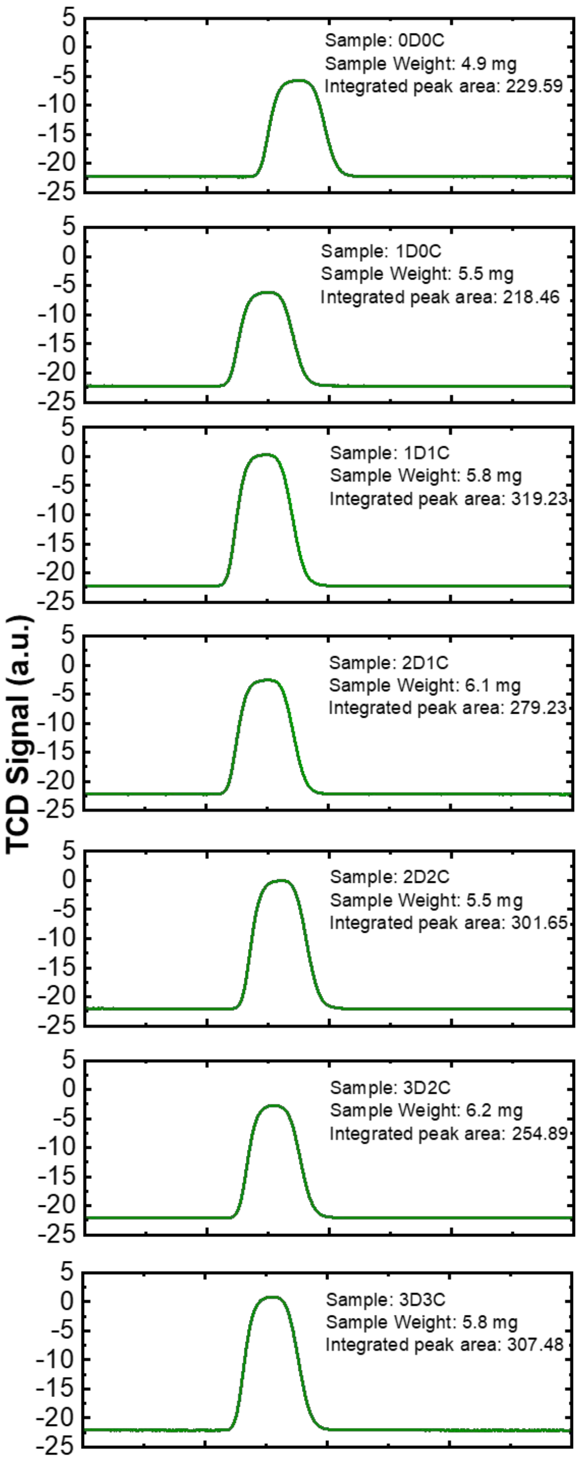

Figure S9. Integrated CO_2 peak area of GC TCD signals from acid titration of electrolytes after cycling cells to different states as indicated (nomenclature: 'XDYC' indicates *X* discharge and *Y* charge half-cycles).

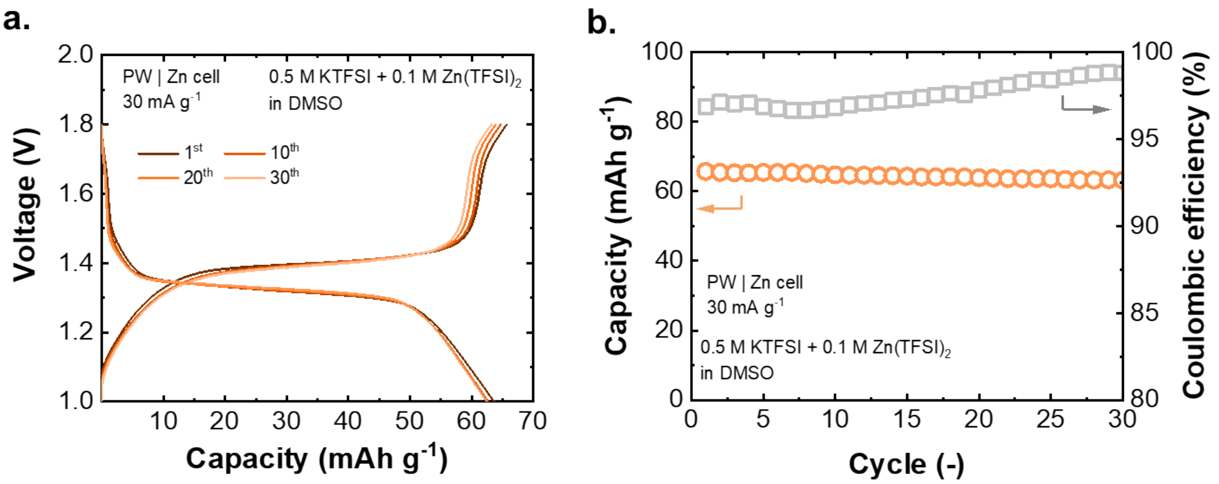

Figure S10. (a) Two-electrode charge and discharge curves for PW/Zn full cell without amine at 30 mA g^{-1} for 30 cycles. (b) Summary of capacity and Coulombic efficiency of PW/Zn full cell without amine for 30 cycles.

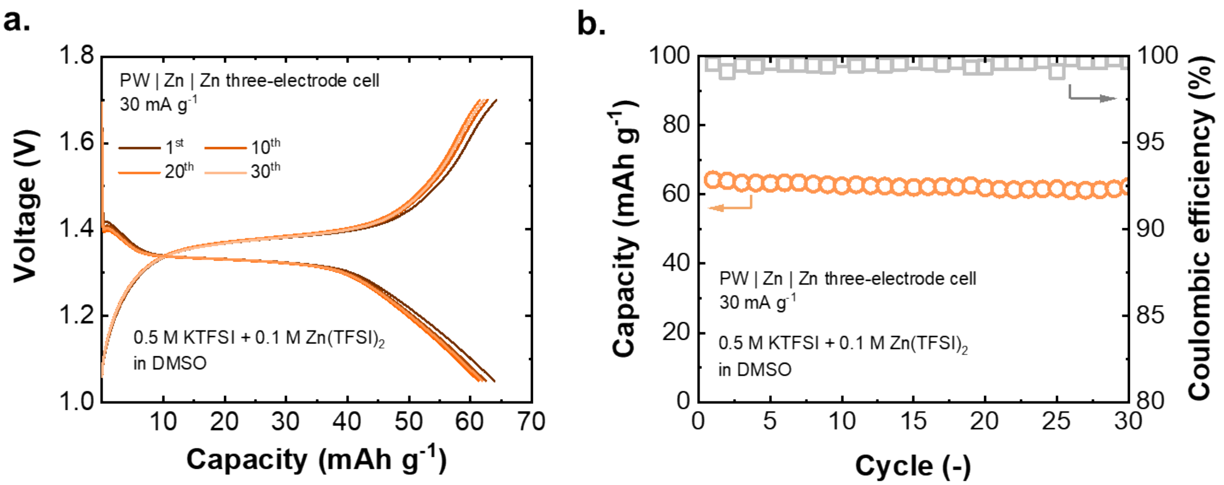

Figure S11. (a) Charge and discharge curves and (b) capacity and CE for the PW three-electrode cell (PW cathode, Zn anode, and Zn reference electrode) without EEA-CO_2 in the electrolyte at 30 mA g^{-1} for 30 cycles.

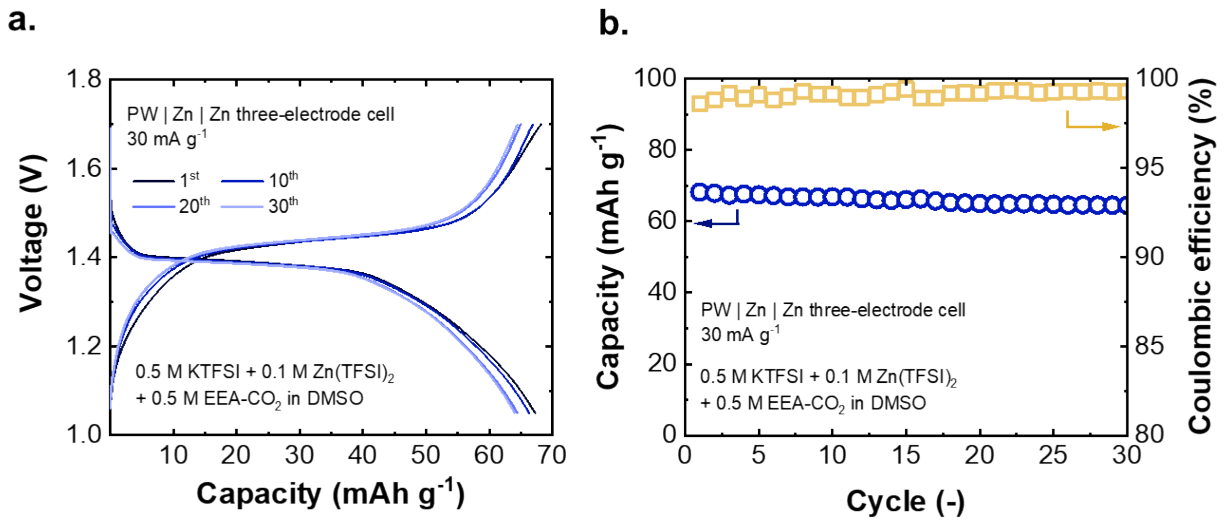

Figure S12. (a) Charge and discharge curves and (b) capacity and CE for the PW three-electrode cell (PW cathode, Zn anode, and Zn reference electrode) with EEA-CO_2 in the electrolyte at 30 mA g^{-1} for 30 cycles.

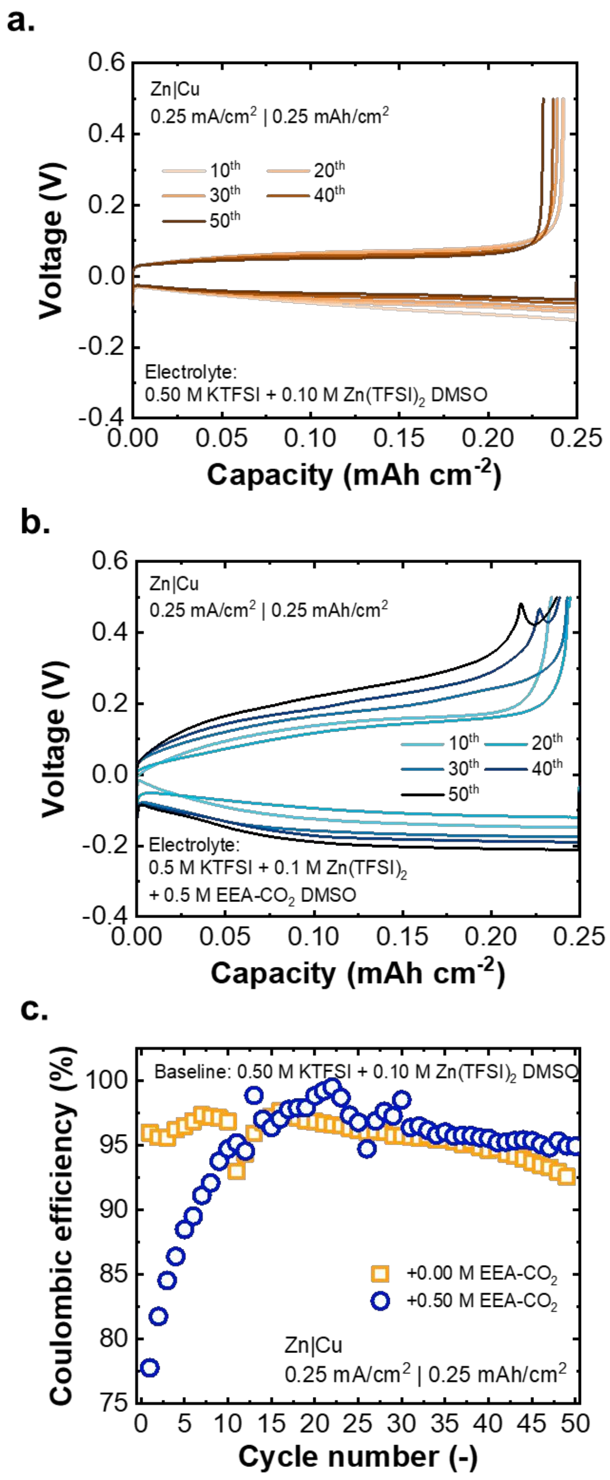

Figure S13. Charge and discharge profiles (current 0.25 mA/cm^2 and capacity 0.25 mAh/cm^2) of two-electrode Zn−Cu cells using 0.50 M KTFSI, 0.10 M Zn(TFSI)$_2$ DMSO electrolyte with (a) 0.0 M EEA-CO_2 and (b) 0.50 M EEA-CO_2. (c) Summary of CEs for 50 cycles.

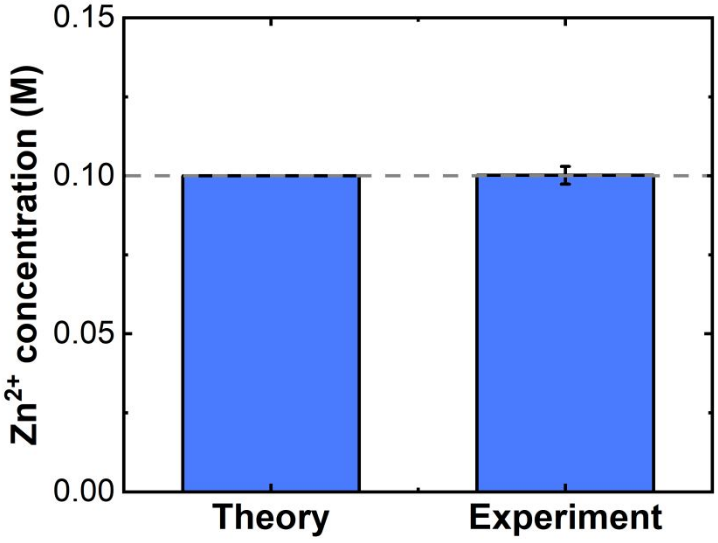

Figure S14. The final electrolyte Zn^{2+} concentration for Zn|Cu cells after 9 cycles (conditions: current 0.25 mA/cm^2, capacity 0.25 mAh/cm^2, and 9 plating + stripping cycles). The data are from the average of 5 independent cells and the error bars indicate standard deviation.

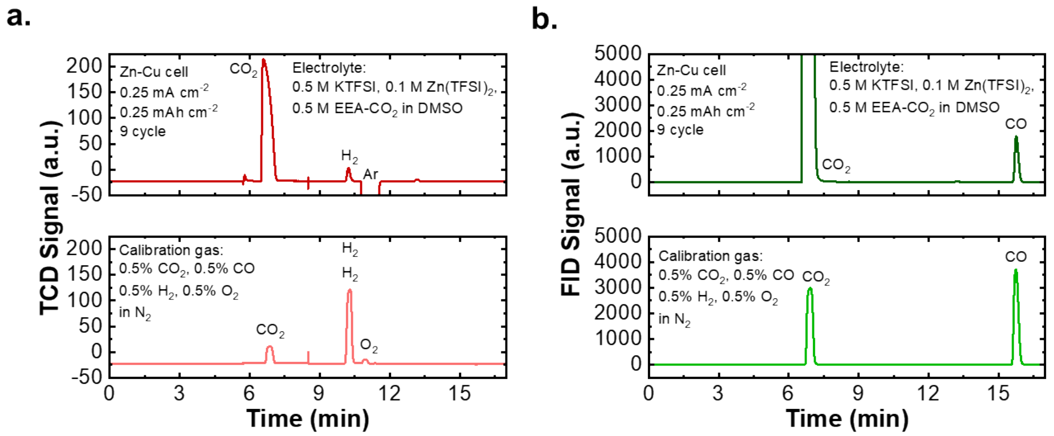

Figure S15. GC (a) TCD and (b) FID signal for the headspace of a Zn/Cu cell (conditions: current 0.25 mA/cm^2, capacity 0.25 mAh/cm^2, and 9 plating + stripping cycles) and calibration gas containing 0.5% H_2. Note that the Zn-Cu cell headspace shows high CO_2 concentration since the cell headspace is purged with CO_2 before cycling.

<u>Figure S15 Discussion</u>

In the electrolyte with EEA-CO_2, carbamic acid and ammonium can participate in the hydrogen evolution reaction (HER), and physically dissolved CO_2 could be potentially reduced to form CO (CO_2RR), leading to capacity loss. To assess possible amounts of H_2 and CO evolution during cell cycling, the headspace gas of Zn/Cu cell containing 0.5 M EEA-CO_2 (conditions: current 0.25 mA/cm^2, capacity 0.25 mAh/cm^2, and 9 plating + stripping cycles) was extracted and injected into the GC to get the headspace gas concentration (Figure S15), which was calculated as follows:

$$\frac{C_{headspace}}{A_{headspace}} = \frac{C_{calibration}}{A_{calibration}} \tag{S7}$$

Where *C* and *A* are the concentration of gas (H_2 or CO) and the integrated peak area (TCD for H_2 or FID for CO) in either the headspace of the cell or calibration gas, respectively. Then, the charge corresponding to HER (Q_{H_2}) and CO_2RR (Q_{CO}) was calculated by

$$Q_{H_2} = \frac{PC_{H_2,headspace}V_{headspace}}{RT} \times nF \tag{S8}$$

$$Q_{CO} = \frac{PC_{CO,headspace}V_{headspace}}{RT} \times nF \tag{S9}$$

Where *P* is the pressure (1 atm), $V_{headspace}$ is the headspace volume of the custom cell (4.0 ml), *R* is the ideal gas constant, *T* is the temperature (25°C), *n* is the number of electron per mol H_2 or CO forming (n = 2 for both cases), *F* is the Faradaic constant (96485 C/mol). $Q_{H_2}$and Q_{CO} are then calculated to be 0.02 C and 0.06 C, respectively, which corresponds to less than 0.01% and 0.04% of the charge loss. Therefore, it could be confirmed that the capacity loss is not from HER and CO_2RR.

Energy Requirement and Areal CO_2 Release Rate Estimation:

Energy requirements were calculated based on the charge and discharge curves of the full cell (Figure S16). The system consumes energy upon charge to reach the CO_2 loaded state; upon cell reversal, energy is released galvanically upon discharge while CO_2 is released. The energy consumption for one cycle is the energy difference between charging and discharging the cell. As for the total amount of CO_2 released, the total amount of Zn^{2+} swing was calculated based on the capacity of the cell assuming that every Zn^{2+} could modulate 1.5 CO_2 release (this assumption is based the CO_2 loading change between 2D1C and 2D2C in Figure 5b). Using the charge and discharge curves from Figure S16 at different rates, the energy consumption for cation-swing process were estimated and tabulated in Table S4.

Table S4. Estimated energy consumption for the cation-swing process under different current density.

Current rate ($mA\ g^{-1}$)	Current density ($mA\ cm^{-2}$)	Energy consumed during charging ($J\ g^{-1}$ PW)	Energy released during discharging ($J\ g^{-1}$ PW)	CO_2 released ($mmol\ g^{-1}$ PW)	Estimated energy consumption ($kJ/mol\ CO_2$)
30	0.12	351	311	1.81	22.1
50	0.20	345	294	1.74	29.6
75	0.30	328	277	1.66	30.8
100	0.40	321	263	1.58	36.5
125	0.50	307	247	1.50	39.4

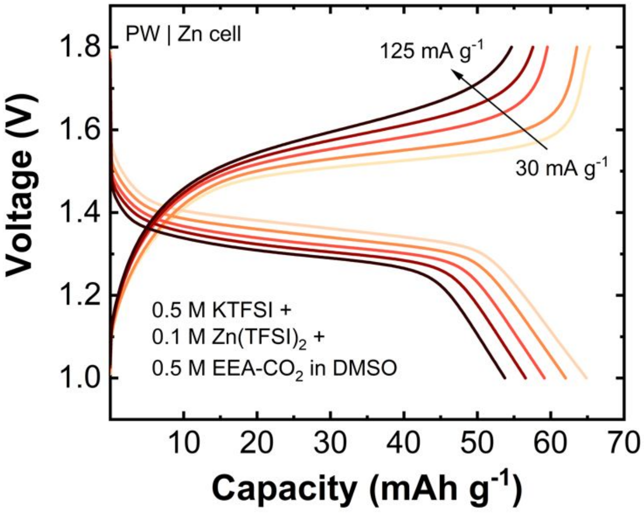

Figure S16. Charge and discharge curves for PW/Zn full cell with amine at 30, 50, 75, 100, and 125 mA g^{-1} (PW loading is 6.93 mg on 15 mm carbon paper for this specific data).

Discussion of Implementing Cation-Swing Process under Post-Combustion Capture Conditions:

For this proof-of-concept work, the cation-swing process operates under fixed (100%) CO_2 partial pressure conditions, which is how the energy requirement for separation was obtained. However, in practice, a CO_2 separation process would be required to capture from a dilute CO_2 stream and release at a higher partial pressure. Therefore, this section provides an estimation of the electrode capacity-to-electrolyte ratios and the increased energy cost to implement such a process compared to those under constant CO_2 partial pressure conditions. The below analysis uses 0.18 bar CO_2 partial pressure as the dilute stream, which is close to post-combustion capture conditions, and 1 bar CO_2 released pressure. Note that the CO_2 solubilities in DMSO are 0.023 and 0.138 M under these pressures, respectively.[5,6]

1. Electrode capacity-to-electrolyte ratios

For CO_2 to release at pressure, the amount of CO_2 must be sufficiently high to exceed the physically dissolved CO_2 in the electrolyte and drive CO_2 into the headspace. Under the operating conditions described above, the solution already has an initial 0.023 M of CO_2 physically dissolved from the previous equilibration at lower partial pressure during the capture step. In this case, the amount of CO_2 modulation in solution, driven electrochemically, must exceed 0.138 M – 0.023 M = 0.115 M. This value can be further converted to electrode charge and yields 3.1 mAh/mL of electrolyte assuming 100% cation conversion efficiency. Note that the above calculation assumes that amines do not substantially alter the physical solubility of DMSO.

In our specific case, we used 250 μL of electrolyte and 0.5 M amine concentration and measured a ~0.15 mol CO_2/mol amine loading delta, which corresponds to 0.075 M change of CO_2 (with respect to the solution volume) and 2.0 mAh/mL. Therefore, to advance the cell design to be suitable for real applications, it is necessary to increase the electrode-to-electrolyte mass ratios and/or identify cathodes with higher capacities for the weak Lewis acid cation, which may include higher-capacity conversion-type electrodes or other intercalation materials in future work. Based on the estimation above, a ~53% increase in electrode capacity would be necessary for practical application, which is not out of reach given that PW materials are far from optimized in terms of capacity compared to other possible available materials or electrode reactions.

2. Increase in energy cost

In considering the energy cost when operating between two different partial pressures, we first summarize the contributions to the energy requirement:

$$\text{Energy cost per mol } CO_2 \text{ (kJ/mol)} = \frac{E_{charge} - E_{discharge}}{N_{CO_2,\,amine} - \Delta N_{CO_2,\,dissolved}} = \frac{(V_{charge} - V_{discharge})Q}{v_{electrolyte}(C_{CO_2,\,amine} - \Delta C_{CO_2,\,dissolved})}$$

where E_{charge} is the energy consumption for cell charge (kJ), $E_{discharge}$ is the energy recovered from cell discharge (kJ), V_{charge} and $V_{discharge}$ are the charge and discharge voltages, respectively, and Q is the electrode capacity (assumed for simplicity to be equal for charge and discharge, *i.e.*, 100% Coulombic efficiency). Meanwhile, $N_{CO_2,\,amine}$ is the amount of CO_2 released from amine (mol), and $\Delta N_{CO_2,\,dissolved}$ is the difference of the amounts of CO_2 dissolved in the solvent between the capture and released partial pressures (mol). Additionally, $v_{electrolyte}$ is the electrolyte volume (L), $C_{CO_2,\,amine}$ is the concentration of CO_2 released from amine (M), and $\Delta C_{CO_2,\,dissolved}$ is the difference of the CO_2 solubilities in the solvent between the capture and released partial pressures (M). The denominator reflects the fact that, when releasing at higher partial pressure than that of the inlet stream, the CO_2 physical solubility is higher due to the higher headspace

partial pressure, and must be overcome by releasing excess CO_2. Therefore, higher electrical work would be required for the same amount of actually released CO_2. Alternatively but equivalently, for the same electrical work, the amount of separated CO_2 must be discounted, identically increasing the per-CO_2 separation cost.

As an example, we assume that the electrolyte volume, electrode capacity Q, and V_{charge} and $V_{discharge}$ are the same as those determined under the constant (100%) CO_2 partial pressure used in this work for simplicity of the estimation. Under these assumptions, (1) the total amount of CO_2 released from the amine ($N_{CO_2, amine}$), directly determined from Q, and (2) the total electrical energy cost per cycle under constant CO_2 pressure (E_{charge} - $E_{discharge}$) would be the same. However, the amount of CO_2 released to the atmosphere (the denominator of the above equation) would be less for reasons noted above. Effectively, then, greater total charge Q is required to yield CO_2 that actually leaves solution and can be flushed out of the headspace, which indeed increases the energy requirement. Also, this increase in energy cost would depend on the CO_2 partial pressures of the dilute stream and amine concentrations. Therefore, for various conditions, we can calculate the increase in energetic cost as shown in Table 1 below assuming the maximum CO_2 released with the cation-swing mechanism is half of the amine concentration, representing the full loading window accessible in DMSO.

Table S5. Percentage of increased energy cost with CO_2 partial pressures in dilute stream and different amine concentrations assuming CO_2 released partial pressure of 1 bar (solubility of CO_2 in DMSO under 1 bar CO_2 is 0.138 M).

Amine concentration (mol/L electrolyte)	$C_{CO_2, amine}$ (mol/L electrolyte)	CO_2 partial pressure in dilute stream (bar)	CO_2 solubilities in DMSO under dilute stream (M)	$\Delta C_{CO_2, dissolved}$ (mol/L electrolyte)	CO_2 released at 1 bar (mol/L electrolyte)[a]	Percentage of increased energy cost (%)[b]
0.5	0.25	0.07	0.009	0.129	0.121	107%
0.5	0.25	0.11	0.014	0.124	0.126	98%
0.5	0.25	0.18	0.023	0.115	0.135	85%
1.0	0.50	0.18	0.023	0.115	0.385	30%
1.5	0.75	0.18	0.023	0.115	0.635	18%

[a] CO_2 released at 1 bar (mol/L electrolyte) = $C_{CO_2, amine} - \Delta C_{CO_2, dissolved}$

[b] Percentage of increased energy cost (%) = $\left(\frac{C_{CO_2, amine}}{C_{CO_2, amine} - \Delta C_{CO_2, dissolved}} - 1\right) \times 100\%$

The energy cost can be significant for relatively low amine concentration, but decreases with higher values because there is higher CO_2 released compared to what can be dissolved. It is also important to note that the above estimation does not account for the differences in pumping requirements in the process, overpotentials in the cell operation, and differences in amine speciation under lower CO_2 partial pressure, which could further affect the energy cost but requires more research to elucidate in full, and is beyond the scope of this work. However, this first-order analysis is sufficient to illustrate how future optimizations might be directed towards increasing amine concentration and using solvents with lower CO_2 solubility to reduce this energy penalty.

Reference:

1. He, G. & Nazar, L. F. Crystallite size control of Prussian White analogues for nonaqueous potassium-ion batteries. *ACS Energy Lett.* **2**, 1122–1127 (2017).

2. Khurram, A., He, M. & Gallant, B. M. Tailoring the discharge reaction in Li-CO_2 batteries through incorporation of CO_2 capture chemistry. *Joule* **2**, 2649–2666 (2018).

3. Khurram, A., Yan, L., Yin, Y., Zhao, L. & Gallant, B. M. Promoting amine-activated electrochemical CO_2 conversion with alkali salts. *J. Phys. Chem. C* **123**, 18222–18231 (2019).

4. Kortunov, P. V, Siskin, M., Baugh, L. S. & Calabro, D. C. In-situ nuclear magnetic resonance mechanistic studies of carbon dioxide reactions with liquid amines in non-aqueous systems: evidence for the formation of carbamic acids and zwitterionic species. *Energy Fuels* **29**, 5940–5966 (2015).

5. Jitaru, M. Electrochemical carbon dioxide reduction-Fundamental and applied topics. *J. Chem. Tech. and Metall.* **42**, 333–344 (2007).

6. Hua, L. Thermodynamic model of solubility for CO_2 in dimethyl sulfoxide. *Phys. Chem. Liquids* **47**, 296–301 (2009).
