# Supplementary material for: Dual Salt Cation-Swing Process for Electrochemical CO2 Separation
Source: ACS Cent Sci. 2023 Aug 30;9(9):1750–7. doi: 10.1021/acscentsci.3c00692 (PMC10540289; doi:10.1021/acscentsci.3c00692)
Supplement: Supplementary file 2 — oc3c00692_si_002.pdf [file oc3c00692_si_002.pdf]

oc-2023-00692y.R1

Name: Peer Review Information for "Dual Salt Cation-Swing Process for Electrochemical CO_2 Separation"

First Round of Reviewer Comments

Reviewer: 1

Comments to the Author

This work explores a very interesting new approach for electrochemical carbon capture, which reversibly modulates the CO_2 loading on liquid amines in organic solvents by changing the identity of the Lewis acid cations in the system. The underlying mechanism of the proposed electrochemical cation swing process was analyzed carefully with NMR-based speciation studies, gas measurements, and calorimetry. As proof of concept, a dual-ion cell with a K^+ intercalation electrode and a Zn electrode was developed to demonstrate the feasibility of modulating the cation identity in amine-based electrolytes. The work is of high quality, and I recommend publication after addressing the following minor comments.

(1) The Coulombic efficiencies of initial cycles of Zn plating-stripping were lower in the presence of EEA (Figure S12). Was Zn lost to solution in the form of Zn^{2+} or afforded side products on the electrode surface?

(2) The change in CO_2 loading during dual-ion cycling was confirmed via acid titration. Was the data provided? I am curious if the authors can detect changes in headspace CO_2 pressure using the setup shown in Figure S6.

(3) It is understandable that as proof-of-concept work, the CO_2 modulation capacity is limited by the capacity of the electrode. It can be a good idea to discuss the electrode capacity required for the practical deployment of this process, considering the CO_2 solubility in DMSO under different headspace CO_2 concentrations. This can be particularly important if we want to release CO_2 at pressure.

(4) Some recent publications on electrochemical carbon capture via direct redox of organic sorbent molecules can be included, such as Nature Energy, 2022, 7, 1065–1075. The citation on neutral red (ref 23) is actually a pH swing mechanism.

Reviewer: 2

Comments to the Author

Kuo et al. demonstrate that an electrochemically driven cation-swing process can modulate CO_2 capture and release via reversible carbamic acid-to-carbamate conversion. The study is well written and the energetic analysis using microcalorimetry is an especially useful contribution. I recommend publication after two points are addressed:

1. It is common practice in the literature, but somewhat misleading, to cite an energetic cost for CO2 separation for a process that captures/releases CO2 from/to a constant-pCO2 environment (100% CO2 in this case). In practice, one would capture and concentrate CO2 from a dilute stream and release it at a higher partial pressure/purity, as shown here (DOI: 10.1038/s41467-022-29791-7). The authors should at least comment on the feasibility of running such a process with their cation-swing cell, and how much of an increase in energetic cost might be expected.

2. The initial discharge capacity for Zn|PW cell in Figure 5a appears to be 45 mAh/g, not 65 mAh/g as written in the text. This apparent discrepancy should be clarified.

Author's Response to Peer Review Comments:

Dear Dr. Editor,

Thank you for forwarding the reviewer comments on our manuscript entitled, “Dual Salt Cation-Swing Process for Electrochemical CO2 Separation”. We were pleased to see that both Reviewers are supportive of publication in ACS Central Science subject to appropriate revisions. We found the Reviewers’ comments very helpful and have been able to address them in the revised manuscript, submitted here for your consideration.

As requested, we are returning to you a separate response letter that includes point-to-point response to the Reviewers comments, a manuscript file (with changes highlighted), and an SI file (with changes highlighted).

Thank you in advance for your handling of our revised manuscript.

On behalf of all authors,

Sincerely,

Betar M. Gallant

Detailed Response to Reviewer Comments

Peer-Reviewer #1 (Comments to the Author):

This work explores a very interesting new approach for electrochemical carbon capture, which reversibly modulates the CO_2 loading on liquid amines in organic solvents by changing the identity of the Lewis acid cations in the system. The underlying mechanism of the proposed electrochemical cation swing process was analyzed carefully with NMR-based speciation studies, gas measurements, and calorimetry. As proof of concept, a dual-ion cell with a K^+ intercalation electrode and a Zn electrode was developed to demonstrate the feasibility of modulating the cation identity in amine-based electrolytes. The work is of high quality, and I recommend publication after addressing the following minor comments.

Author response: We thank the reviewer for the supportive and constructive comments about our manuscript and for indicating its suitability to be accepted with revision.

1. The Coulombic efficiencies of initial cycles of Zn plating-stripping were lower in the presence of EEA (Figure S12). Was Zn lost to solution in the form of Zn^{2+} or afforded side products on the electrode surface?

Author response: We thank the reviewer for the good question. To further investigate the Coulombic inefficiencies (CIE) of initial cycles, we have conducted additional experiments using Zn/Cu cells with 0.5 M EEA-CO_2 in the electrolyte. The following experimental procedure was added on p. S12 of SI:

For analysis of Coulombic inefficiencies of Zn|Cu cells with 0.5 M EEA-CO_2 in the initial cycles, the cells were opened after 9 cycles (conditions: current 0.25 mA/cm^2, capacity 0.25 mAh/cm^2, and 9 plating + stripping cycles). The separators were soaked in 1 ml DMSO for 3 h, and the solution was then filtered with a syringe filter to remove any solids. The filtrate was mixed with 3 wt% HNO_3 solution to a final composition of 10 vol% of DMSO and 90 vol% of 3 wt% HNO_3 solution as the ICP sample for quantification of final Zn^{2+} concentration in the electrolyte.

We have added the following figure on p. S18 of SI:

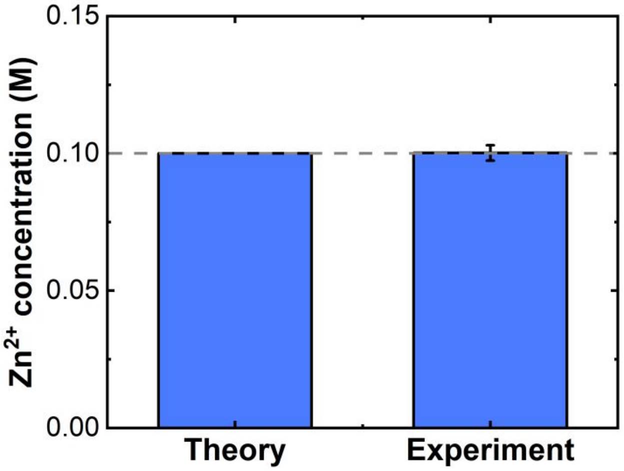

Figure S14. The final electrolyte Zn^{2+} concentration for Zn|Cu cells after 9 cycles (conditions: current 0.25 mA/cm^2, capacity 0.25 mAh/cm^2, and 9 plating + stripping cycles). The data are from the average of 5 independent cells and the error bars indicate standard deviation.

We have added the following discussion regarding the result of Figure S14 on p. 17 of manuscript. We also took the opportunity to further clarify the entire discussion regarding capacity fading in addition to CE, so we copy the entire paragraph below for the convenience of the reviewer:

Finally, the long-term cycling performance of the PW/Zn cell was evaluated (Figure 5c). With amines and CO_2 in the cell, 94.2% of capacity was retained after 30 cycles (Figure 5d), which is only slightly lower than that of cells without amine (96.2%, Figure S10; three-electrode cell measurements in Figure S11–S12). Given that these cells utilize a Zn metal anode which acts as a quasi-infinite reservoir, the observed losses arise at the PW cathode. To further understand the intrinsic Zn cyclability in the two electrolytes, two-electrode Zn/Cu cells were also examined (Figure S13). The Zn plating/stripping CEs of the amine-containing cell were initially lower than without amine, but eventually approached and even exceeded that with amine, yielding 96.4% from the 20th to the 50th cycle compared to 95.1% without. A higher degree of Zn plating/stripping polarization was also observed with amine present. The initial CE discrepancy of Zn/Cu cells with and without amine was not due to Zn loss (corrosion) to the solution during cycling, given that the Zn^{2+} concentration in the electrolyte remained constant at 0.10 M after cycling with amines present (Figure S14). We could also confirm that no H_2 and CO evolution, such as from possible parasitic decomposition of amines, occurred during Zn cycling by extracting the headspace gas from the cell for GC gas quantification (Figure S15). Therefore, the different initial CE values can be attributed to initial electrode conditioning differences to form the SEI on the Cu current collector, which is common for metal plating/stripping reactions. Given the above observations, the small capacity and cycling differences without and with amine in full cells is hypothesized to arise from cell polarization differences arising primarily at the Zn anode, which can lead to capacity slippage of PW in the full cell configuration and will be the subject of focused future work, including development of optimized cycling protocols. Regardless, the good Zn CE and overall PW/Zn cell cycling performance indicates reasonable stability suitable for further development.

2. The change in CO_2 loading during dual-ion cycling was confirmed via acid titration. Was the data provided? I am curious if the authors can detect changes in headspace CO_2 pressure using the setup shown in Figure S6.

Author response: We thank the reviewer for pointing this out and we have added the GC raw data and the integrated CO_2 peak area in New Figure S9, as shown below. As for detecting changes in headspace CO_2, unfortunately, our setup is currently unable to detect headspace CO_2 pressure. It is a very good suggestion and we will take this into consideration for future study. We also want to point out that the change of CO_2 pressure in the current cell is expected to be small given the electrode capacity, which is not well-balanced with the headspace volume in this particular cell. Given current electrode capacity (0.4–0.5 mAh), the theoretical amount of CO_2 released is 0.18–0.23 mL based on the Ideal Gas law. The headspace volume of the cell is 4 ml, and thus the change of the pressure will only be 0.05 atm. Therefore, optimization of these parameters would be needed in the future for pressure change detection.

We have added the following figure on p. S14 of SI:

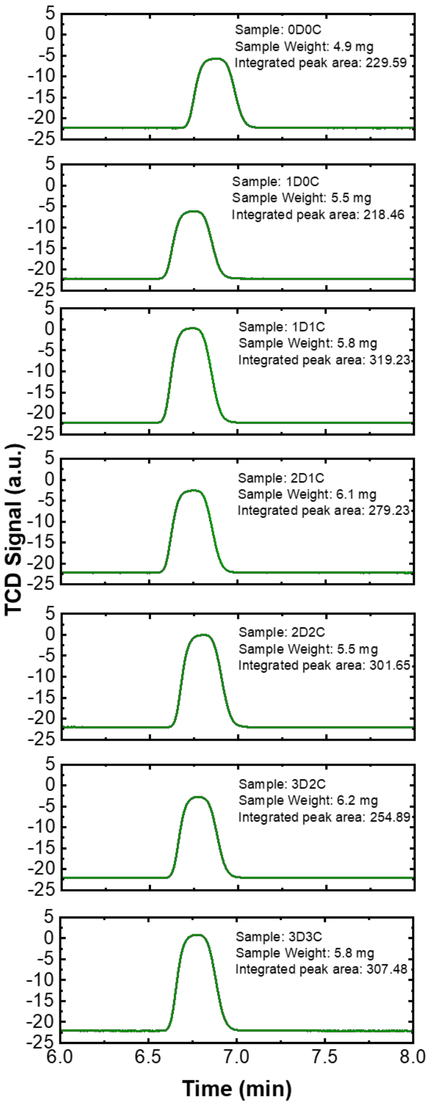

New Figure S9. Integrated CO_2 peak area of GC TCD signals from acid titration of electrolytes after cycling cells to different states as indicated (nomenclature: 'XDYC' indicates *X* discharge and *Y* charge half-cycles).

3. It is understandable that as proof-of-concept work, the CO_2 modulation capacity is limited by the capacity of the electrode. It can be a good idea to discuss the electrode capacity required for the practical deployment of this process, considering the CO_2 solubility in DMSO under different headspace CO_2 concentrations. This can be particularly important if we want to release CO_2 at pressure.

Author response: Thank you for the excellent question. We have added the following analysis on p. S21 of SI to discuss the target CO_2 loading modulation in practical applications for capture of CO_2 from a dilute stream and release at a higher partial pressure:

Discussion of Implementing Cation-Swing Process under Post-Combustion Capture Conditions:

For this proof-of-concept work, the cation-swing process operates under fixed (100%) CO_2 partial pressure conditions, which is how the energy requirement for separation was obtained. However, in practice, a CO_2 separation process would be required to capture from a dilute CO_2 stream and release at a higher partial pressure. Therefore, this section provides an estimation of the electrode capacity-to-electrolyte ratios and the increased energy cost to implement such a process compared to those under constant CO_2 partial pressure conditions. The below analysis uses 0.18 bar CO_2 partial pressure as the dilute stream, which is close to post-combustion capture conditions, and 1 bar CO_2 released pressure. Note that the CO_2 solubilities in DMSO are 0.023 and 0.138 M under these pressures, respectively.[5,6]

1. Electrode capacity-to-electrolyte ratios
   For CO_2 to release at pressure, the amount of CO_2 must be sufficiently high to exceed the physically dissolved CO_2 in the electrolyte and drive CO_2 into the headspace. Under the operating conditions described above, the solution already has an initial 0.023 M of CO_2 physically dissolved from the previous equilibration at lower partial pressure during the capture step. In this case, the amount of CO_2 modulation in solution, driven electrochemically, must exceed 0.138 M – 0.023 M = 0.115 M. This value can be further converted to electrode charge and yields 3.1 mAh/mL of electrolyte assuming 100% cation conversion efficiency. Note that the above calculation assumes that amines do not substantially alter the physical solubility of DMSO.
   In our specific case, we used 250 μL of electrolyte and 0.5 M amine concentration and measured a ~0.15 mol CO_2/mol amine loading delta, which corresponds to 0.075 M change of CO_2 (with respect to the solution volume) and 2.0 mAh/mL. Therefore, to advance the cell design to be suitable for real applications, it is necessary to increase the electrode-to-electrolyte mass ratios and/or identify cathodes with higher capacities for the weak Lewis acid cation, which may include higher-capacity conversion-type electrodes or other intercalation materials in future work. Based on the estimation above, a ~53% increase in electrode capacity would be necessary for practical application, which is not out of reach given that PW materials are far from optimized in terms of capacity compared to other possible available materials or electrode reactions.

We have added the following reference on p. S23 of SI:

6. Hua, L. Thermodynamic model of solubility for CO_2 in dimethyl sulfoxide. *Phys. Chem. Liquids* **47**, 296–301 (2009).

and the following on p. 17 of manuscript (please note that these edits also incorporate changes in response to Question 1 from Reviewer 2):

Based on the above data obtained under pure (100%) CO_2 partial pressure, the electrical energy of the cation-swing capture-release process reported herein was calculated to be ~22-39 kJ/mol CO_2 at an equivalent areal current of 0.1–

0.5 mA cm^{-2} (calculations in the experimental section). This range represents a minimum energy estimate, which will be larger if capture is conducted at lower partial pressures as relevant for practical applications. It was estimated that an additional minimum ~50% increase in electrode capacity and ~85% increase in energy cost per mol CO_2 separated would be required to capture CO_2 from a dilute stream (0.18 bar) and release at 100% purity (atmospheric pressure, 1 bar) for the same amine concentration and voltages used herein. These increases are attributed to the need to overcome the difference in CO_2 solubilities in DMSO under different partial pressures. On the other hand, increasing the amine concentration, and/or decreasing the CO_2 solubility of the solvent by moving beyond DMSO are effective strategies to limit this additional energy requirement to within ~20–30% (see discussion in SI).

4. Some recent publications on electrochemical carbon capture via direct redox of organic sorbent molecules can be included, such as Nature Energy, 2022, 7, 1065–1075. The citation on neutral red (ref 23) is actually a pH swing mechanism.

Author response: We thank the reviewer for pointing this out and we have added the suggested literature in the introduction. Also, the reviewer is of course correct that neutral red (ref 23) is a pH swing mechanism, and we have corrected the placement of this reference (ref 24) in the introduction section.

On p. 3:

Salient examples of electrochemical CO_2 separation processes include electrochemically mediated amine regeneration (EMAR),[17-19,] direct redox of organic sorbent molecules (e.g. quinones[14,20,21], sp^2-nitrogen base[22,23], *etc.*), and pH swing[16,24,25] methods achieved through water electrolysis or use of bipolar membranes.

On p. 23:

[23] Li, X., Zhao, X., Liu, Y., Hatton, T. A. & Liu, Y. Redox-tunable Lewis bases for electrochemical carbon dioxide capture. *Nat. Energy* **7**, 1065–1075 (2022).

[24] Seo, H. & Hatton, T. A. Electrochemical direct air capture of CO_2 using neutral red as reversible redox-active material. *Nat. Commun.* **14**, 1–11 (2023).

Peer-Reviewer #2 (Comments to the Author):

Kuo et al. demonstrate that an electrochemically driven cation-swing process can modulate CO_2 capture and release via reversible carbamic acid-to-carbamate conversion. The study is well written and the energetic analysis using microcalorimetry is an especially useful contribution. I recommend publication after two points are addressed.

Author response: We thank the reviewer for the constructive suggestions about our manuscript and the recommended modifications, which we have addressed in our revision as detailed below.

1. It is common practice in the literature, but somewhat misleading, to cite an energetic cost for CO_2 separation for a process that captures/releases CO_2 from/to a constant-pCO_2 environment (100% CO_2 in this case). In practice, one would capture and concentrate CO_2 from a dilute stream and release it at a higher partial pressure/purity, as shown here (DOI: 10.1038/s41467-022-29791-7). The authors should at least comment on the feasibility of running such a process with their cation-swing cell, and how much of an increase in energetic cost might be expected.

Author response: Thank you for the good question. First, we wish to note that we provided an estimate of the operating conditions (capacity-to-electrolyte ratios) required to capture/release CO_2 at different partial pressures in response to Reviewer 1, Comment 3, assuming DMSO as solvent. For the estimation of the increase in energetic cost, we have added the following discussion on p. S21 of the SI:

2. Increase in energy cost

In considering the energy cost when operating between two different partial pressures, we first summarize the contributions to the energy requirement:

$$\text{Energy cost per mol } CO_2 \text{ (kJ/mol)} = \frac{E_{charge} - E_{discharge}}{N_{CO_2,\,amine} - \Delta N_{CO_2,\,dissolved}} = \frac{(V_{charge} - V_{discharge})Q}{V_{electrolyte}(C_{CO_2,\,amine} - \Delta C_{CO_2,\,dissolved})}$$

where E_{charge} is the energy consumption for cell charge (kJ), $E_{discharge}$ is the energy recovered from cell discharge (kJ), V_{charge} and $V_{discharge}$ are the charge and discharge voltages, respectively, and Q is the electrode capacity (assumed for simplicity to be equal for charge and discharge, *i.e.*, 100% Coulombic efficiency). Meanwhile, $N_{CO_2,\,amine}$ is the amount of CO_2 released from amine (mol), and $\Delta N_{CO_2,\,dissolved}$ is the difference of the amounts of CO_2 dissolved in the solvent between the capture and released partial pressures (mol). Additionally, $v_{electrolyte}$ is the electrolyte volume (L), $C_{CO_2,\,amine}$ is the concentration of CO_2 released from amine (M), and $\Delta C_{CO_2,\,dissolved}$ is the difference of the CO_2 solubilities in the solvent between the capture and released partial pressures (M). The denominator reflects the fact that, when releasing at higher partial pressure than that of the inlet stream, the CO_2 physical solubility is higher due to the higher headspace partial pressure, and must be overcome by releasing excess CO_2. Therefore, higher electrical work would be required for the same amount of actually released CO_2. Alternatively but equivalently, for the same electrical work, the amount of separated CO_2 must be discounted, identically increasing the per-CO_2 separation cost.

As an example, we assume that the electrolyte volume, electrode capacity Q, and V_{charge} and $V_{discharge}$ are the same as those determined under the constant (100%) CO_2 partial pressure used in this work for simplicity of the estimation. Under these assumptions, (1) the total amount of CO_2 released from the amine ($N_{CO_2,\,amine}$), directly determined from

Q, and (2) the total electrical energy cost per cycle under constant CO_2 pressure (E_{charge} - $E_{discharge}$) would be the same. However, the amount of CO_2 released to the atmosphere (the denominator of the above equation) would be less for reasons noted above. Effectively, then, greater total charge Q is required to yield CO_2 that actually leaves solution and can be flushed out of the headspace, which indeed increases the energy requirement. Also, this increase in energy cost would depend on the CO_2 partial pressures of the dilute stream and amine concentrations. Therefore, for various conditions, we can calculate the increase in energetic cost as shown in Table 1 below assuming the maximum CO_2 released with the cation-swing mechanism is half of the amine concentration, representing the full loading window accessible in DMSO.

Table S5. Percentage of increased energy cost with CO_2 partial pressures in dilute stream and different amine concentrations assuming CO_2 released partial pressure of 1 bar (solubility of CO_2 in DMSO under 1 bar CO_2 is 0.138 M).

Amine concentration (mol/L electrolyte)	$C_{CO_2, \text{amine}}$ (mol/L electrolyte)	CO_2 partial pressure in dilute stream (bar)	CO_2 solubilities in DMSO under dilute stream (M)	$\Delta C_{CO_2, \text{dissolved}}$ (mol/L electrolyte)	CO_2 released at 1 bar (mol/L electrolyte)[a]	Percentage of increased energy cost (%)[b]
0.5	0.25	0.07	0.009	0.129	0.121	107%
0.5	0.25	0.11	0.014	0.124	0.126	98%
0.5	0.25	0.18	0.023	0.115	0.135	85%
1.0	0.50	0.18	0.023	0.115	0.385	30%
1.5	0.75	0.18	0.023	0.115	0.635	18%

[a] CO_2 released at 1 bar (mol/L electrolyte) = $C_{CO_2, \text{amine}} - \Delta C_{CO_2, \text{dissolved}}$

[b] Percentage of increased energy cost (%) = $\left(\frac{C_{CO_2, \text{amine}}}{C_{CO_2, \text{amine}} - \Delta C_{CO_2, \text{dissolved}}} - 1\right) \times 100\%$

The energy cost can be significant for relatively low amine concentration, but decreases with higher values because there is higher CO_2 released compared to what can be dissolved. It is also important to note that the above estimation does not account for the differences in pumping requirements in the process, overpotentials in the cell operation, and differences in amine speciation under lower CO_2 partial pressure, which could further affect the energy cost but requires more research to elucidate in full, and is beyond the scope of this work. However, this first-order analysis is sufficient to illustrate how future optimizations might be directed towards increasing amine concentration and using solvents with lower CO_2 solubility to reduce this energy penalty.

The following sentence is added on p. 17 of manuscript as the pointer for the above discussion in SI:

Based on the above data obtained under pure (100%) CO_2 partial pressure, the electrical energy of the cation-swing capture-release process reported herein was calculated to be ~22-39 kJ/mol CO_2 at an equivalent areal current of 0.1–0.5 mA cm^{-2} (calculations in the experimental section). This range represents a minimum energy estimate, which will be larger if capture is conducted at lower partial pressures as relevant for practical applications. It was estimated that an additional minimum ~50% increase in electrode capacity and ~85% increase in energy cost per mol CO_2 separated would be required to capture CO_2 from a dilute stream (0.18 bar) and release at 100% purity (atmospheric pressure, 1 bar) for the same amine concentration and voltages used herein. These increases are attributed to the need to overcome the difference in CO_2 solubilities in DMSO under different partial pressures. On the other hand, increasing the amine concentration, and/or decreasing the CO_2 solubility of the solvent by moving beyond DMSO are effective strategies to limit this additional energy requirement to within ~20–30% (see discussion in SI).

2. The initial discharge capacity for Zn|PW cell in Figure 5a appears to be 45 mAh/g, not 65 mAh/g as written in the text. This apparent discrepancy should be clarified.

Author response: We thank the reviewer for pointing this out, and that was indeed unintentionally unclear. The initial cell discharge capacity is 43 mAh g^{-1} and 65 mAh g^{-1} is the second discharge capacity. In theory, PW has the formula of $K_2Fe^{II}[Fe^{II}(CN)_6]$, where both Fe have a +2 charge. Thus, the electrode should normally be fully potassiated upon cell assembly, only allowing charge as the first step. However, after synthesis, the contact of PW particles with water during the washing procedure partially oxidizes the Fe^{2+} to Fe^{3+}, and the particle color turns from white to blue, indicating that it is partially converted into Prussian blue ($KFe^{III}[Fe^{II}(CN)_6]$). As a result, we can begin by discharging the cell before conducting a full charge, during which the portion of the cathode material that is converted to Prussian blue was able to be re-potassiated. However, since the conversion is only partial, the first discharge would not be able to achieve the full capacity (65 mAh/g), but only 45 mAh/g as shown in Original Figure 5a.

We have addressed this with the following edit. On p. 14:

The cell, which was capacity-limited by the PW electrode, demonstrated an initial discharge capacity of 43 mAh g^{-1} and a second discharge capacity of 65 mAh g^{-1}. The initial discharge capacity, corresponding to potassiation of PW, is normally not present in stoichiometrically synthesized PW cathodes, but was accessible here due to partial reversible oxidation of PW during synthesis.
